# STUB1 stabilizes sirtuin 6 via K63-linked ubiquitination to promote NRF2 deacetylation for enhanced ferroptosis resistance after spinal cord injury

**DOI:** 10.1093/burnst/tkag047

**Published:** 2026-07-14

**Authors:** Zhuanghui Wang, Wu Ye, Jiaxing Wang, Xuhui Ge, Yufeng Zhu, Wei Liu, Qinghong Ma, Weihua Cai, Yuluo Rong

**Affiliations:** Department of Spine Surgery, The Affiliated Jiangning Hospital with Nanjing Medical University, Nanjing, Jiangsu 210000, China; Department of Orthopedics, The First Affiliated Hospital of Nanjing Medical University, Nanjing, Jiangsu 210000, China; Department of Orthopedics, The First Affiliated Hospital of Nanjing Medical University, Nanjing, Jiangsu 210000, China; Department of Orthopedics, Taizhou People’s Hospital of Nanjing Medical University, Taizhou School of Clinical Medicine, Nanjing Medical University, Taizhou 225300, China; Department of Orthopedics, Changzheng Hospital, Second Military Medical University, Shanghai 200003, China; Department of Orthopedics, The First Affiliated Hospital of Nanjing Medical University, Nanjing, Jiangsu 210000, China; Department of Orthopedics, Changzheng Hospital, Second Military Medical University, Shanghai 200003, China; Department of Spine Surgery, The Affiliated Jiangning Hospital with Nanjing Medical University, Nanjing, Jiangsu 210000, China; Department of Orthopedics, The First Affiliated Hospital of Nanjing Medical University, Nanjing, Jiangsu 210000, China; Department of Orthopaedics, The First Affiliated Hospital of University of Science and Technology of China, Division of Life Sciences and Medicine, University of Science and Technology of China, Hefei, Anhui 230001, China; State Key Laboratory of Trauma and Chemical Poisoning, Army Medical University, Chongqing 400038, China

**Keywords:** Spinal cord injury, STUB1/SIRT6/NRF2, Ferroptosis, Neuron, Ubiquitination, Neuronal ferroptosis

## Abstract

**Background:**

Spinal cord injury (SCI) is a severe traumatic condition with limited treatments. Ferroptosis, an iron-dependent type of controlled cell death, participates in SCI pathology. Nonetheless, the molecular mechanisms governing ferroptosis after SCI remain incompletely understood. Therefore, this study aims to elucidate the regulatory role of SIRT6 in neuronal ferroptosis post-SCI, identify its upstream modulator and downstream target, and evaluate the therapeutic potential of targeting this pathway for functional recovery.

**Methods:**

Ferroptosis markers were assessed in SCI mouse models and primary neurons. Sirtuin 6 (SIRT6) expression was regulated via lentiviral vectors and adeno-associated viral vectors. Protein interactions were identified by immunoprecipitation coupled with mass spectrometry and co-immunoprecipitation. Ubiquitination assays were conducted using ubiquitin mutants. Nuclear transcription factor erythroid 2-related factor 2 knockout (NRF2-KO) mice were used for *in vivo* validation. Functional recovery was evaluated by Basso Mouse Scale score, rotarod test, footprint analysis, and electromyography.

**Results:**

SIRT6 expression was downregulated following SCI and during erastin-induced ferroptosis. SIRT6 overexpression inhibited ferroptosis and triggered functional recovery, while SIRT6 suppression exacerbated these effects. Mechanistically, SIRT6 interacted with and deacetylated NRF2, promoting its nuclear translocation and antioxidant gene activation. STIP1 homology and U-box containing protein 1 (STUB1) was identified as an upstream regulator that physically interacted with SIRT6 via its U-box domain. STUB1 stabilized SIRT6 by promoting K63-linked ubiquitination, protecting it from proteasomal degradation. STUB1 overexpression inhibited ferroptosis and improved functional recovery, effects that were abolished by SIRT6 knockdown or NRF2-KO.

**Conclusions:**

The STUB1/SIRT6/NRF2 axis represents a novel regulatory pathway that suppresses neuronal ferroptosis and promotes functional recovery post-SCI. Targeting this axis may offer a prospective therapeutic strategy for SCI.

## Graphical Abstract

**Graphical Abstract:**
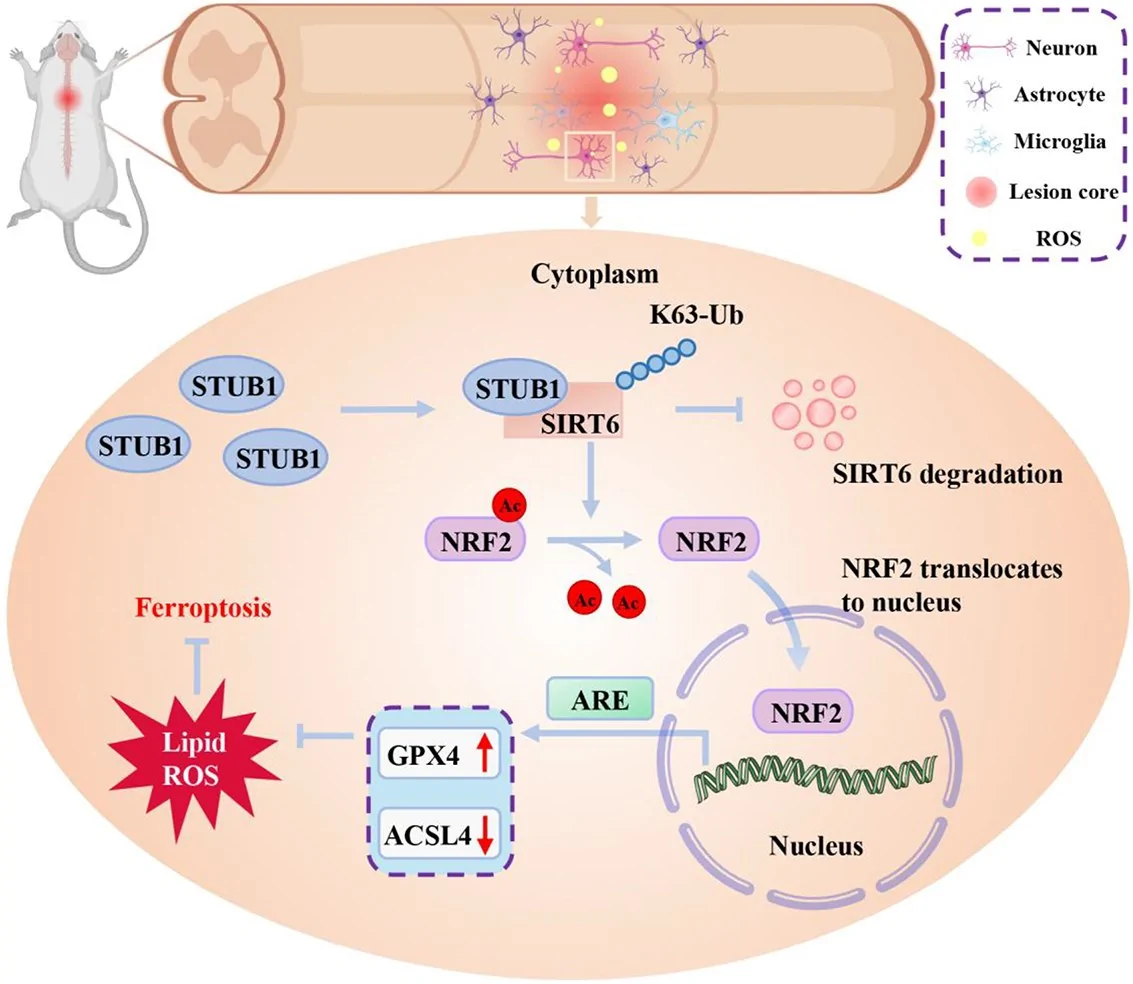

## Highlights

- Sirtuin 6 (SIRT6) is downregulated after spinal cord injury (SCI) and suppresses neuronal ferroptosis.
- SIRT6 deacetylates nuclear transcription factor erythroid 2-related factor 2 (NRF2) to promote its nuclear translocation and antioxidant defense.
- STIP1 homology and U-box containing protein 1 (STUB1) stabilizes SIRT6 via K63-linked ubiquitination, preventing its proteasomal degradation.
- The STUB1/SIRT6/NRF2 axis is a promising therapeutic target for SCI.

## Background

Spinal cord injury (SCI) is a severe traumatic central nervous system disease with high morbidity, disability, mortality, and complications [1]. The incidence of SCI has increased with the rapid development of fast transportation and high-altitude operation in recent years, imposing a heavy public health burden. SCI develops in a two-stage process: primary injury and secondary injury [2]. Primary SCI causes destruction and death of axons and neurons, while secondary injury is associated with the spread of damage and deterioration of the surrounding microenvironment due to pro-inflammatory response [3, 4]. Various neurorestorative treatment strategies are currently used to treat SCI. However, their effects are minimal, and it is imperative to understand the molecular and cellular mechanisms of SCI.

Ferroptosis, a newly discovered cell death mechanism, features iron-mediated overload of lipid reactive oxygen species (ROS), lipid peroxidation, mitochondrial dysfunction, and neuroinflammatory reactions that culminate in cellular and neuronal impairment. It varies from other types of programmed cell death (PCD) in the morphology and biological alterations [5, 6]. As a biochemically different form of PCD, ferroptosis has shown great potential in numerous pathological conditions (e.g. cancer, degenerative diseases, and ischemic disorders) [7, 8]. Our previous research implicated ferroptosis in spinal cord ischemia–reperfusion injury (SCIRI), highlighting autophagy-dependent ferroptosis as a prospective treatment target [9]. The process of ferroptosis can be categorized into these pathways: the canonical GPX4-regulated, the lipid metabolism, and the iron metabolism pathways. Nuclear transcription factor erythroid 2-related factor 2 (NRF2) is a negative controller of ferroptosis, activating the transcription of antioxidant stress genes in the nucleus by targeting antioxidant response elements (ARE) to maintain metabolism and redox homeostasis [10]. Although recent investigations have discovered a relationship between ferroptosis and SCI [11, 12], the detailed mechanism, especially the upstream regulatory targets, remains unknown and requires further research.

Sirtuins are nicotinamide adenine dinucleotide (NAD+)-dependent deacylases that control critical cellular processes in cancer, cardiovascular, and neurodegenerative diseases [13, 14]. Among mammals, seven sirtuin proteins (SIRT1–SIRT7) have been identified, each exhibiting unique N- and C-terminal structural features, distinct subcellular localization patterns, and specific functional roles [15]. SIRT6, the mammalian homolog of yeast silent information regulator 2 (Sir2), has been reported to regulate lifespan as well as several fundamental processes, including metabolism, telomere maintenance, deoxyribonucleic acid (DNA) repair, and neuronal health [16–18]. Several proteins that participate in specific biological processes, including SMAD2 and RUNX2, have been identified as SIRT6 substrates [19, 20]. Another study revealed that oxidative stress-induced activation of miR-34a reduces SIRT6 expression and its anti-aging functions [21]. Given its role in stress resistance, it is noteworthy that SIRT6 is a main regulator of oxidative stress. Specifically, SIRT6 has been shown to coactivate the transcription factor NRF2, a master negative regulator of ferroptosis, to safeguard cells from oxidative damage [22], illustrating a potential connection between SIRT6 and the ferroptotic cell death pathway. Although SIRT6 has been thoroughly studied, its effects and mechanism in SCI, particularly in neuronal cell death, remain unknown.

In the current study, based on the established connection between SIRT6 and NRF2, we investigated whether SIRT6 has a function in neuronal ferroptosis after SCI. Here, SIRT6 is a functional histone deacetylase that is downregulated in SCI and is associated with severe neuronal cell ferroptosis. Our outcomes illustrated that SIRT6 suppresses ferroptosis by the interaction with and positively regulating NRF2/ARE, protecting neurons against SCI. Moreover, we demonstrate that the E3 ligase STIP1 homology and U-box containing protein 1 (STUB1) is responsible for SIRT6 protein protection during ferroptosis. This investigation illustrates an innovative function of the STUB1/SIRT6/NRF2 axis in the onset of neuronal ferroptosis. Therefore, targeting STUB1/SIRT6/NRF2 axis may be a prospective approach for SCI.

## Methods

### Design of animal experiments

No significant differences in body temperature, weight, or food intake were detected among groups prior to the experiment. To investigate the temporal expression of SIRT6 and its STUB1-mediated regulation in ferroptosis after SCI, mice were randomly assigned into experimental groups. In the first set, sham (*n* = 6) and SCI (*n* = 30, subdivided into Days 1, 3, 7, 14, 28 post-injury, *n* = 6 each) were used to measure SIRT6 levels, ferroptosis markers (ACSL4, GPX4), iron content, lipid peroxidation, and glutathione (GSH) via western blot, quantitative polymerase chain reaction (qPCR), immunofluorescence, and biochemical assays. In the second set, intraspinal adeno-associated viruses (AAVs) injections targeting neurons were performed in four groups (SCI + AAV-Con+ AAV-shCon, SCI + AAV-STUB1+ AAV-shCon, SCI + AAV-Con+ AAV-shSIRT6, and SCI + AAV-STUB1 + AAV-shSIRT6; *n* = 24 each), alongside an NRF2 knockout (NRF2-KO) cohort (SCI + AAV-Con, SCI + AAV-SIRT6, and SCI + AAV-STUB1; *n* = 24 each). Behavioral assessments were conducted at Days 1, 3, 7, 14, 21, and 28; at endpoints, spinal cord tissues were harvested for western blot, immunofluorescence, dihydroethidium (DHE) staining, and transmission electron microscopy (TEM) to evaluate ferroptosis markers, neuronal survival, axonal regeneration, and mitochondrial morphology.

### Animals and SCI model

NRF2-knockout mice (C57BL/6 background) were sourced from Cyagen Bioscience (Guangzhou, China). The Animal Research Committee of the 1st Affiliated Hospital of the University of Science and Technology, China, gave its approval to all animal procedures [granted number: 2025-N(A)-0128]. SCI models were created via 8-week-old male C57BL/6 mice (Animal Core Facility of the University of Science and Technology, Hefei, China). Anesthesia of mice was conducted with isoflurane inhalation, followed by careful removal of the T8 vertebral lamina to expose the spinal cord. SCI was then induced at the T8 segment using an impactor (Model 68097, RWD, USA) with a striker weight of 5 g dropped from a height of 6.5 cm. Muscle and skin layers were sutured directly post-injury. Postoperatively, manual bladder expression was conducted daily until recovery of reflexive bladder control. The sham surgery group underwent T8 laminectomy only. Ferrostatin-1 (1 mg/kg/day) was given intraperitoneal injection at 7 days pre-SCI and once daily until euthanasia.

### Functional behavioral analysis

Neurological function was assessed in all mice on post-injury Days 1, 3, 7, 14, 21, and 28. Prior to testing, mice were acclimated to the environment for 1 h. Mice were allocated to experimental groups at random via a computer-generated random number table before any surgical procedures. Randomization was performed by an independent researcher who did not participate in animal surgery, behavioral assessments, or data analysis. To ensure baseline homogeneity, body weight, locomotor activity, and rotarod performance were measured before surgery and confirmed to be comparable across all groups. For experiments involving multiple time points or subgroups, separate randomization lists were generated for each cohort. All behavioral evaluations were conducted by testers who were blinded to experimental group allocation, and the data were analyzed under blinded conditions.

#### Basso mouse scale

This scale estimates hindlimb motor function, with a score range of 0 (complete paralysis) to 9 (normal function). Specific parameters assessed include hindlimb joint movement, trunk position/stability, coordination, paw placement, step height, and tail position.

#### Rotarod test

The motor coordination ability of mice was determined using a mouse rotating bar instrument. The mice were placed on a 3 cm-diameter rotating rod and the speed was increased from 0 to 40 revolutions per minute. All mice were examined twice at 20 min intervals, and the average descending latency was calculated.

#### Footprint analysis

The forelimbs and hind legs of mice were dyed blue and red, respectively, and the stride length and width of mice running at a uniform speed were calculated and analyzed.

#### Electromyography test

Motor evoked potentials (MEPs) were recorded using electromyography (EMG) to evaluate spinal cord motor pathway integrity. Electrical stimulation was applied to the rostral spinal cord through a stimulating electrode, while responses were recorded from the hindlimb biceps femoris muscle. The reference electrode was attached to the distal tendon, and a subcutaneous ground electrode was used. MEPs were elicited using single square-wave pulses (0.5 mA, 0.5 ms, 1 Hz), and signal amplitudes were analyzed to assess motor conduction.

### Immunofluorescence staining assays

Anesthetized mice were intracardially perfused with 0.9% saline, followed by paraformaldehyde (PFA; 4% w/v). The injured spinal cord segments were eliminated, fixed in 4% PFA for 24 h, and dehydration was conducted through a graded series of sucrose solutions (15% and 30%). Spinal cord tissue was embedded with OCT embedding agent, and 14 μm longitudinal slices were prepared and kept at −80°C. For spinal cord immunofluorescence staining, frozen sections underwent 10-min fixation in 4% PFA, followed by 10-min permeabilization using 0.3% Triton X-100. After blockage with 5% bovine serum albumin (BSA), sections were immunolabeled with primary antibodies overnight at 4°C (anti-GFAP, anti-NeuN, anti-IBA-1, anti-CD31, anti-SIRT6, and anti-NF200). The sections were exposed to the corresponding conjugated secondary antibodies (AlexaFluor 488 or 594; 1:300; Jackson ImmunoResearch, USA), and the nuclei were counterstained with DAPI (1:1000; Invitrogen, Carlsbad, CA). The immunoreactivity was observed via a fluorescence microscope (Stellaris STED; LEICA, Germany).

### Cell lines and primary neuron culture

For primary neuron culture, the postnatal Day 1 (P1)SPF mice were immersed in 75% alcohol for a few seconds. The skull and perichondrium were dissected after decapitation. After meninges removal, the cerebral cortex was cut into 0.5–1 mm^3^ with ophthalmic scissors, and 0.25% trypsin (Thermo FisherScientific, USA) was added to digest and separate neurons. After the digestion was terminated, the tissue was centrifuged to acquire cell suspensions. The cells were re-suspended in DMEM/F12 (1:1) complete medium (enriched with 10% horse serum and 1% penicillin–streptomycin, USA), and passed through a cell strainer. Cells were cultivated at 1 × 10^6^ cells/ml onto poly-L-lysine (Sigma-Aldrich, USA)-coated culture plates. After 4–6 h incubation, medium was substituted with neurobasal maintenance medium with 2% B27, 1% penicillin–streptomycin, and 1% glutamine.

HEK293T cells were acquired and cultivated in DMEM enriched with 10% fetal bovine serum, 100 U/ml penicillin, and 100 μg/mL streptomycin at 37°C in a 5% CO_2_ atmosphere.

Herein, the reagents consisted of: MG132 (S1748, Beyotime), CHX (HY-12320, MedChemExpress), erastin (SC0224, Beyotime), and Ferrostatin-1 (HY-100579, MedChemExpress).

### Reverse transcription quantitative polymerase chain reaction

Total RNA was extracted from neurons and spinal cord tissue via Trizol ®reagent (Invitrogen, USA) as per the manufacturer’s guidelines. Complementary DNA (cDNA)synthesis was conducted via a reverse transcription system (Toyobo, Japan). SYBR Green PCR Master Mix (Applied Biosystems, USA) was employed for Quantitative Reverse Transcription Polymerase Chain Reaction (qRT-PCR) on ABI 7900 fast real-time PCR system (applied biosystems, USA), and GAPDH acted as the internal control. The 2^−ΔΔCT^ technique was utilized to evaluate the relative expression. These are the primer sequences: SIRT6 primer: F 5′- ATGTCGGTGAATTATGCAGCA-3′ and R 5′-GCTGGAGGACTGCCACATTA-3′. GAPDH primer: F 5′-GGAGAGTGTTTCCTCGTCCC-3′ and R 5′-ATGAAGGGGTCGTTGATGGC-3′.

### Plasmids and lentiviral infection

Transfection was conducted via Lipofectamine 3000 reagent (Invitrogen, USA) as per the manufacturer’s guidelines. The following expression plasmids were constructed by cloning open reading frames (ORFs) into expression vectors with N-terminal Flag or Myc tags (Genebay Biotech, China): Flag-tagged STUB1 (full-length FL 1-303), Flag-tagged STUB1 H260Q mutant, Flag-tagged STUB1 domains (ΔU-box 1-226, ΔTPR 127-303, and U-box 226-303, respectively), and Myc-tagged SIRT6. Plasmids coding for HA-tagged ubiquitin (WT, K6, K11, K27, K29, K33, K48, and K63 mutant) were obtained from Genebay Biotech. Cells were infected with lentivirus at a multiplicity of infection (MOI) of 50, including: control shRNA lentivirus (Lv-shControl), STUB1-targeting shRNA lentivirus (Lv-shSTUB1), SIRT6-targeting shRNA lentivirus (Lv-shSIRT6), NRF2-targeting shRNA lentivirus (Lv-shNRF2), lentivirus empty vector (Lv-Vec), STUB1-overexpressing lentivirus (Lv-STUB1), or SIRT6-overexpressing lentivirus (Lv-SIRT6) (all from Genebay Biotech, China).

### Adeno-associated virus infection

pAAV-PHP.eB-hSyn-STUB1–3 × FLAG-WPRE (AAV-STUB1) and pAAV-PHP.eB-hSyn-SIRT6–3 × FLAG-WPRE (AAV-SIRT6) were constructed (Obio Technology, China) to upregulate the exogenous expression of STUB1 and SIRT6 *in vivo*. pAAV-PHP.eB-hSyn-WPRE (Obio Technology, Shanghai, China) served as negative control (AAV-Con). AAV-PHP.eB vectors for a short hairpin RNA expression directed at SIRT6 (AAV-shSIRT6) or a control hairpin (AAV-shCon) were produced for local SIRT6 depletion *in vivo*. Immediately after SCI, mice received intraspinal injections of the viral vector (5 × 10^9^ vg, 1 μL) at 200 nL/min via a 10 μL Hamilton syringe coupled with a micro-syringe pump controller. Injections were conducted at two sites: 1 mm rostral and 1 mm caudal to the lesion epicenter, at a depth of 1 mm from the dorsal spinal cord surface.

### *In vitro* mechanical scratch injury

The *in vitro* SCI model was established in primary cortical neuron cultures according to published methods [23, 24]. Scratches were made in a 4 × 4 grid pattern (4-mm line spacing) on 24-well culture plates using a 10 μL plastic stylet needle to induce injury in cortical neurons. Untreated cells served as controls.

### Analysis of cell death and cell viability

Cell viability was examined via a Cell Counting Kit-8 (Dojindo, Kumamoto, Japan). Cells were plated in 96-well plates, incubated with the reagent for 2 h at 37°C, and optical density (OD) was assessed at 450 nm via a microplate reader (ELx800; Bio-Tek, USA). Cell death was stained with propidium iodide (PI) staining and analyzed via microscopy.

### Detection of lipid peroxidation, glutathione, and iron content

Lipid peroxidation was assessed via the lipid peroxidation (MDA) Assay Kit (ab118970, Abcam, USA) as per the manufacturer’s instructions. Briefly, thiobarbituric acid (TBA) solution was applied to samples and standards, and then an incubation was conducted at 95°C for 60 min, and cooling was conducted on ice for 10 min, transferred to microplates, and measured with a microplate reader. GSH relative concentration was determined in cell lysates using a GSH Assay Kit (CS0260, Sigma-Aldrich, USA). Iron relative concentration was quantified via an Iron Assay Kit (ab83366, Abcam, USA).

### Reactive oxygen species analysis

We detected ROS levels via an ROS assay kit (Beyotime, China) with 2′,7′-dichlorodihydrofluorescein diacetate (DCFH-DA) as the fluorescent probe, as per the manufacturer’s guidelines, and assessed via a flow cytometer (FACSCalibur; BD Biosciences, USA).

### Dihydroethidium staining

We incubated freshly prepared frozen spinal cord sections with 2 μmol/L of the fluorescent dye DHE (Thermo Fisher Scientific, USA) under light protection for 30 min to estimate the spinal cord oxidative stress level. ImageJ (National Institutes of Health, USA) was utilized to assess the red fluorescence intensity.

### Transmission electron microscopy

Mitochondrial morphology was detected using TEM. Cell samples were treated as follows: First fixed with 0.1 M sodium dimethylarsenate buffer (pH 7.4) with 2.5% glutaraldehyde, and then fixed with 2% aqueous osmium tetroxide solution. Subsequently, staining with 2% uranyl acetate solution was conducted for 2 h, followed by dehydrating with layer ethanol (30%–100%) and propylene oxide in turn. Finally, after embedding cells, we prepared ultrathin sections for visualization under Tecnai electron microscope produced by FEI Corporation (Hillsboro, USA).

### Antibodies

Immunoblotting utilized the following antibodies: GAPDH (1:2000, Servicebio), SIRT6 (1:2000, Proteintech), STUB1 (1:2000, Abclonal), NRF2 (1:2000, Santa Cruz Biotechnology), GPX4 (1:5000, Abcam), ACSL4 (1:10000, Abcam), 4-HNE (1:1000, Abcam), as well as Flag-Tag, Myc-Tag, and HA-Tag (All 1:2000, Cell Signaling Technology). For immunofluorescence, antibodies included: GFAP (1:1000, Abcam; 1:400, Cell Signaling Technology), SIRT6 (1:100, Santa Cruz Biotechnology), IBA-1 (1:200, Servicebio), NeuN (1:200, Abcam), CD31 (1:300, Abcam), and STUB1 (1:400, Santa Cruz Biotechnology).

### Western blot analysis

Following cell lysis with radioimmunoprecipitation assay (RIPA) buffer (Beyotime, China), equal protein quantities were electrophoresed by sodium dodecyl sulfate polyacrylamide gel electrophoresis and transferred to polyvinylidene fluoride (PVDF) membranes. After blockage of membranes with 5% BSA, they were incubated overnight at 4°C with primary antibodies and then with horseradish peroxidase (HRP)-conjugated secondary antibodies (1:10000; Jackson ImmunoResearch, USA) for 2 h at room temperature. Enhanced chemiluminescence was applied for target band detection. We conducted a semi-quantitative analysis of protein band intensities via ImageJ.

### Immunoprecipitation

We prepared neuronal whole-cell lysates via a weak RIPA lysis buffer and pre-cleared them with 50% protein A/G agarose beads (Santa Cruz Biotechnology, USA) for 1 h at 4°C. Subsequently, an incubation of 500 μL of the lysate was conducted with 2 μg of primary antibody overnight at 4°C. Precipitation of immune complexes was conducted by applying protein A/G agarose beads, and incubation was conducted for 2 h at 4°C with constant agitation. We collected the beads and rinsed them 5 times with RIPA lysis buffer. We eluted bound proteins by boiling in 1× loading buffer, then assessed them by immunoblotting.

### Immunoprecipitation with mass spectrometry (IP/MS)

Following isolation of total neuronal proteins, IP was conducted via specific primary antibodies with protein A/G-agarose beads (Santa Cruz Biotechnology, USA). The resulting immunoprecipitates were purified and subsequently assessed by mass spectrometry. The immunoprecipitated proteins were trypsin-digested. We separated the resulting peptides by nano-liquid chromatography and ionized them via nano-electrospray ionization. MS analysis was conducted on a mass spectrometer (Thermo Fisher Scientific, USA).

### Statistical analyses

Data are reported as mean ± SD from at least three independent biological replicates. GraphPad Prism (v8) (GraphPad Software, USA) was utilized for statistical analyses. We assessed data distribution via the Shapiro–Wilk test before all multiple-group comparisons. For two-group comparisons, we employed an unpaired two-tailed Student’s *t-*test. We employed one-way ANOVA with Tukey’s post hoc test for analyses of multiple groups. In trials estimating variables across numerous conditions, two-way ANOVA and then Bonferroni correction were employed. P < 0.05 was deemed significant.

## Results

### Ferroptosis happens post-SCI, and the SIRT6 expression is decreased *in vivo*

**Figure 1:**
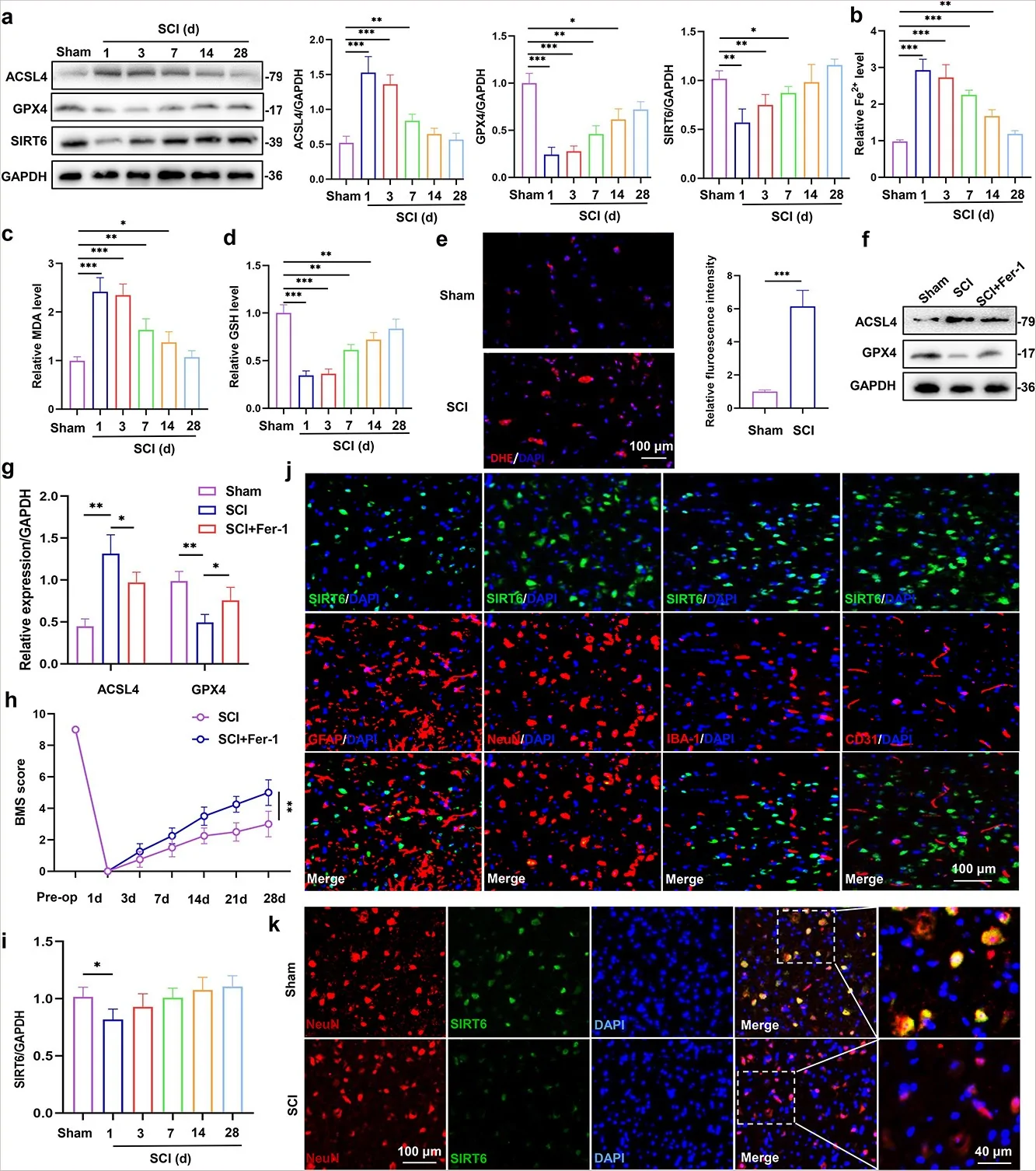
Ferroptosis occurs after SCI and the expression of SIRT6 is decreased *in vivo*. (**a**) Representative immunoblots showing levels of SIRT6 and ferroptosis-associated proteins in injured spinal cord at days 1, 3, 7, 14, and 28 after SCI (left). Quantification of relative levels of indicated protein (right, n = 6). (**b**-**d**) The relative values of Fe^2+^ concentrations, MDA and GSH in injured spinal cord were determined (n = 6). (**e**) DHE staining was used to detect ROS levels in the injured spinal cord at 3 days postinjury (*n* = 6) (Scale bar: 100 μm). (**f**-**g**) Representative western blot showing the expression of ferroptosis-associated proteins in the injured spinal cord at 3 days postinjury. Quantification of levels of indicated protein (*n* = 3). (**h**) BMS score results of indicated groups after SCI (*n* = 6). (**i**) Expression of SIRT6 mRNA in injured spinal cord examined by qPCR (*n* = 6). (**j**) Immunofluorescence analysis of the co-localization of SIRT6 with neurons (NeuN^+^), astrocytes (GFAP^+^), macrophages/microglia (IBA-1^+^), and endothelial cells (CD31^+^) (Scale bar: 100 μm). (**k**) The expression of SIRT6 after SCI was analyzed by immunofluorescence at 3 days postinjury (Scale bar: 100 μm; 40 μm). ^*^p <0.05; ^*^^*^p <0.01; ^*^^*^^*^p <0.001. *SCI* spinal cord injury, *ACSL4* Acyl-CoA synthetase long chain family member 4, *GPX4* Glutathione peroxidase 4, *SIRT6* Sirtuin 6, *MDA* malondialdehyde, *GSH* glutathione, *DHE* Dihydroethidium, *ROS* reactive oxygen species, *BMS* basso mouse scale, *GFAP* glial fibrillary acidic protein, *NeuN* neuronal nuclei, *Iba1* ionized calcium-binding adapter molecule 1

To develop novel SCI treatments, we focused on neuronal ferroptosis. Given their central roles as execution factors, we measured the levels of core proteins ACSL4 and GPX4 at numerous time points post-SCI *in vivo*. The positive regulator of ferroptosis, ACSL4, was elevated post-SCI and peaked on the first- and third-day post-injury, while GPX4 decreased to a minimum on the first day (Figure 1a), illustrating that ferroptosis happens in the early stage of SCI. We assessed the relative levels of Fe^2+^, MDA, and GSH to confirm the production of hydrogen peroxide phospholipid. GSH in SCI levels decreased, while MDA and Fe^2+^ increased significantly and peaked on the first and third days (Figure 1b–d). Furthermore, DHE intensity increased in the damaged spinal cord (Figure 1e). Therefore, SCI can lead to a significant overload of lipid peroxidation products, a typical feature recognizing ferroptosis from other types of PCD.

To examine the function of ferroptosis suppression in SCI, mice were subsequently administered Ferrostatin-1 (Fer-1), a specific and potent ferroptosis inhibitor. Fer-1 treatment elevated GPX4 expression while reducing ACSL4 level (Figure 1f and g). Functional behavioral analysis, including Basso mouse scale (BMS) score, rotarod test, and footprint analysis, demonstrated that ferroptosis inhibition promoted functional recovery after SCI (Figures 1h and S1a–c).

SIRT6 participates in metabolism, telomere maintenance, DNA repair, and neuronal health. We first detected SIRT6 mRNA expression using qPCR (Figure 1i) to assess the potential function of SIRT6 in the injured spinal cord. The SIRT6 gene expression was down-regulated in early-stage SCI mice comparative to the Sham-operated group, with the lowest expression on Day 1. SIRT6 protein levels were also significantly reduced after SCI, with the lowest expression on the first day (Figure 1a). Several cell types, including astrocytes (GFAP^+^), macrophages/microglia (IBA-1^+^), neurons (NeuN^+^), and endothelial cells (CD31^+^) are found in the spinal cord. Earlier investigations have indicated that SIRT6 is primarily expressed in neuronal cells throughout the brain [25]. We investigated further SIRT6 distribution in the spinal cord, and immunofluorescence analysis illustrated that SIRT6 was primarily located in NeuN^+^ neuronal cells but not in other types of cells (Figures 1j and S1d). Subsequently, we conducted double immunofluorescence staining for SIRT6 and the neuronal marker NeuN in spinal cord tissue further demonstrated a reduction in SIRT6 levels within NeuN^+^ cells during the early stages of SCI (Figures 1k and S1e). These outcomes illustrate that SIRT6 may have a vital function in the pathogenesis after SCI.

### SIRT6 is decreased in erastin-triggered ferroptosis of primary neurons

**Figure 2:**
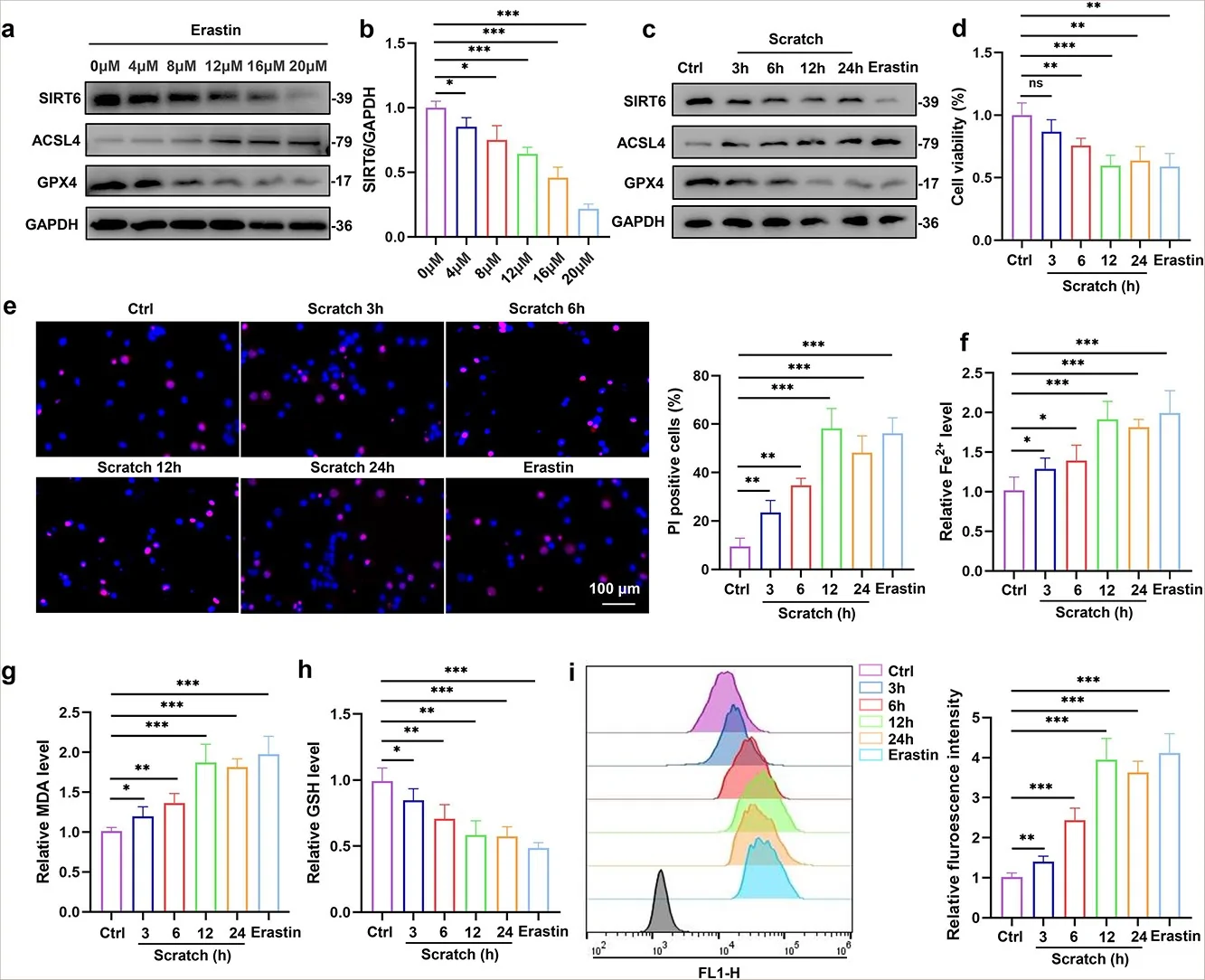
SIRT6 is decreased in erastin-induced ferroptosis of primary neurons. (**a**-**b**) Erastin exposure decreases SIRT6 protein expression (left). Quantification of relative levels of indicated protein (right, *n* = 6). (**c**-**i**) The neuronal cells were treated with Erastin (12 µM) for 24h. (c) The expression of ferroptosis-associated proteins in indicated groups was detected by western blot analysis (*n* = 6). (d) Cell viability after scratch injury was analyzed by CCK-8 (*n* = 6). (e) PI staining was used to detect cell survival after scratch treatment (*n* = 6) (Scale bar: 100 μm). (f-i) The relative values of Fe^2+^ concentrations, MDA, GSH and ROS in indicated groups were measured (*n* = 6). ^*^p <0.05; ^*^^*^p <0.01; ^*^^*^^*^p <0.001. *SIRT6* Sirtuin 6, *ACSL4* Acyl-CoA synthetase long chain family member 4, *GPX4* glutathione peroxidase 4, *PI* propidium iodide, *MDA* malondialdehyde, *GSH* glutathione, *ROS* reactive oxygen species

SCI was induced *in vitro* using an established mechanical scratch injury model [23]. The ferroptosis activator erastin was employed as a positive control. Cells were extracted from the neonatal mouse cerebral cortex and treated with classical mechanical scratch injury and different concentrations of erastin after 7 days of culture. A 24 h erastin treatment of cells decreased SIRT6 expression (Figure 2a and b). Mechanical injury significantly decreased SIRT6 expression (Figure 2c), similar to erastin treatment.

Furthermore, ACSL4 was significantly overexpressed in the mechanical injury and erastin treatment groups, peaking at 12 h in the mechanical injury group, while GPX4 expression was decreased (Figure 2c). CCK8 and PI staining also displayed a significant decrease in viable cells (Figure 2d and e). The main events in ferroptosis are Fe^2+^ overload, lipid peroxidation, and GSH depletion. Mechanical injuring neurons significantly decreased GSH activity, while MDA and Fe^2+^ levels were significantly elevated *in vitro* (Figure 2f–h). We then examined the change in mechanical injury-induced ROS levels and found that ROS levels increased in these neurons (Figure 2i). We used Fer-1 to rescue the primary neurons subjected to mechanical scratch injury to clarify further the critical function of ferroptosis during SCI *in vitro*. Resembling the outcomes *in vivo*, Fer-1 treatment significantly decreased cell death as assessed by CCK8 assay and reversed ferroptosis events (Figure S2a–e). These findings are consistent with SIRT6’s role in regulating ferroptosis in primary neurons.

### SIRT6 upregulation declines erastin-triggered neuronal cytotoxicity and lipid peroxidation

**Figure 3:**
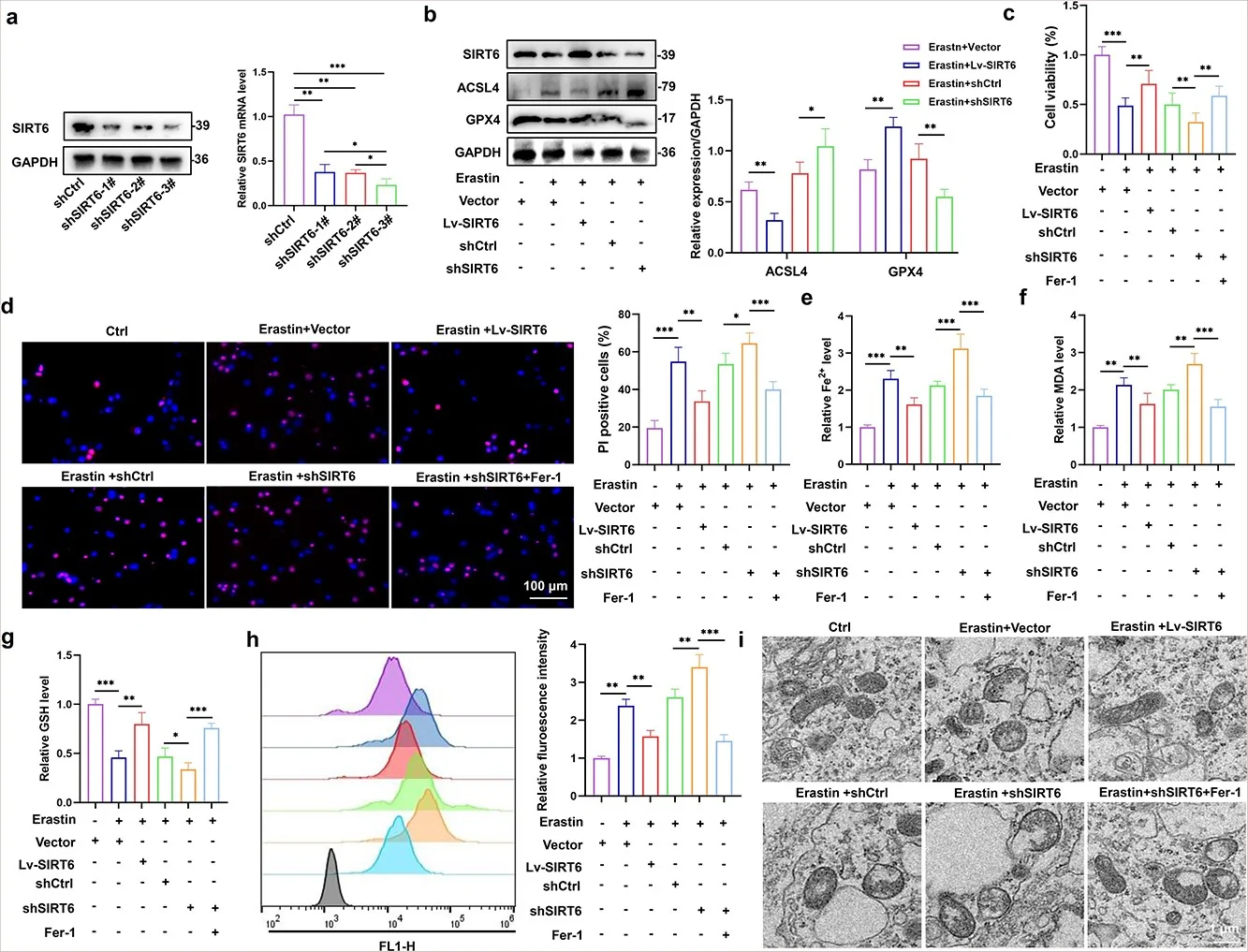
Upregulation of SIRT6 decreases erastin-induced neuronal cytotoxicity and lipid peroxidation. (**a**) Western blot analysis of SIRT6 expression after transfection in primary neurons (left, *n* = 3). Quantification of SIRT6 mRNA levels (right, *n* = 3). (**b**) Western blot analysis was used to detect the expression of ferroptosis-associated proteins (left). Quantification of relative levels of related protein (right, *n* = 6). (**c**) Cell viability in indicated groups analyzed by CCK-8 (*n* = 6). (**d**) PI staining was used to detect cell survival after Erastin treatment (*n* = 6) (Scale bar: 100 μm). (**e**-**h**) Fe^2+^ concentrations, MDA, GSH and ROS were measured after Erastin treatment (*n* = 6). (**i**) Representative TEM images show shrunken mitochondria and outer membrane rupture upon exposure to Erastin (*n* = 6) (Scale bar:1 μm). ^*^p <0.05; ^*^^*^p <0.01; ^*^^*^^*^p <0.001. *SIRT6* Sirtuin 6, *ACSL4* Acyl-CoA synthetase long chain family member 4, *GPX4* Glutathione peroxidase 4, *GAPDH* Glyceraldehyde-3-phosphate Dehydrogenase, *PI* propidium iodide, *MDA* malondialdehyde, *GSH* glutathione, *ROS* reactive oxygen species, *TEM* transmission electron microscopy

We utilized primary neurons to examine the function of SIRT6 as a ferroptosis regulator. SIRT6 expression was modulated using lentiviruses: SIRT6 overexpression (Lv-SIRT6) and SIRT6 knockdown (shSIRT6). Among three tested shSIRT6 sequences, one demonstrated optimal knockdown efficiency (Figure 3a). Western blot analysis revealed that erastin treatment elevated ACSL4 levels while reducing GPX4 expression. Notably, SIRT6 overexpression attenuated the erastin-induced effects, decreasing ACSL4 and increasing GPX4 expression. Conversely, SIRT6 knockdown significantly exacerbated these effects, further suppressing GPX4 and promoting ACSL4 expression. (Figure 3b). Exogenous SIRT6 abolished the partial viability inhibition of erastin-treated cells measured by CCK-8 assay and PI staining. In contrast, shSIRT6-treated neurons decreased cell viability further (Figure 3c and d). Furthermore, we discovered that SIRT6 overexpression reversed ferroptosis events, including Fe^2+^ accumulation, GSH, MDA content, and ROS production (Figure 3e–h). In contrast, SIRT6 knockdown aggravated these effects, while Fer-1 inhibited ferroptosis and the damage caused by SIRT6 suppression (Figure 3c–h). TEM studies showed that erastin treatment caused mitochondrial shrinkage, outer mitochondrial membrane rupture, and light vacuole formation, which are characteristic changes of ferroptosis. SIRT6 overexpression prevented these changes (Figure 3i). These findings suggest that SIRT6 overexpression reduced erastin-induced neuronal cytotoxicity and lipid peroxidation.

### Upregulation of SIRT6 attenuates ferroptosis and triggers functional recovery post-SCI

**Figure 4:**
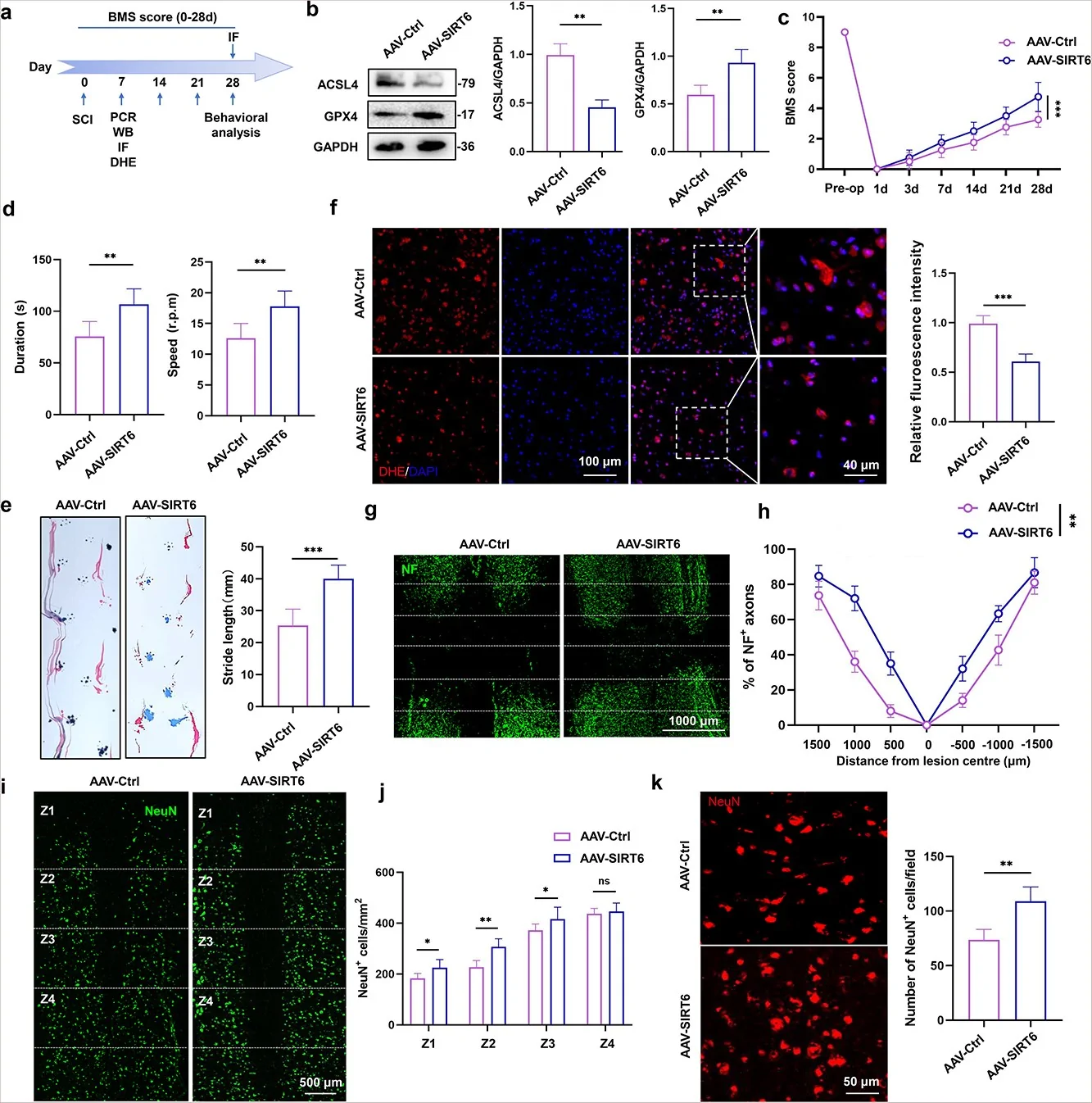
Overexpression of SIRT6 attenuates ferroptosis and promotes functional recovery after SCI. (**a**) Schematic diagram showing experimental design. (**b**) Representative western blot showing the expression of ferroptosis-associated proteins in the injured spinal cord at 7 days postinjury (left). Quantification of relative levels of related protein (right, *n* = 6). (**c**) BMS score results after SCI (*n* = 6). (**d**-**e**) Rotarod test and footprint results at 28 days postinjury (*n* = 6). (**f**) DHE staining was used to detect ROS levels in the injured spinal cord at 7 days postinjury (*n* = 6) (Scale bar: 100 μm; 40 μm). (**g**) Representative immunofluorescence images for NF in injured spinal cords at 28 days postinjury (Scale bar: 1000 μm). (**h**) Quantitative analysis of NF positive area at various distances from SCI lesion center as percentage of total area of distant uninjured axons (*n* = 3). (**i**) Representative immunofluorescence images of NeuN^+^ neurons in Z1-Z4 regions adjacent to lesion core at 28 days postinjury (Scale bar: 500 μm). (**j**) Quantification of NeuN^+^ neurons in Z1-Z4 regions adjacent to lesion core (*n* = 3). (**k**) Representative enlarged immunofluorescence images of NeuN^+^ neurons in the damaged spinal cord at 28 days postinjury (n =6) (Scale bar: 50 μm). ^*^p <0.05; ^*^^*^p <0.01; ^*^^*^^*^p <0.001. *SIRT6* Sirtuin 6, *ACSL4* Acyl-CoA synthetase long chain family member 4, *GPX4* glutathione peroxidase 4, *BMS* basso mouse scale, *SCI* spinal cord injury, *DHE* Dihydroethidium, *ROS* reactive oxygen species, *NF200* neurofilament 200, *NeuN* neuronal nuclei

We injected neuronal cell-specific adeno-associated virus (AAV-PHP.eB-hSyn) carrying SIRT6 sequence (AAV-SIRT6) into injured mice immediately after SCI to validate that SIRT6 upregulation triggers functional recovery and inhibits ferroptosis. Western blotting illustrated that AAV-SIRT6 injection overexpressed SIRT6 in both Sham-operated and SCI mice (Figure S3a). Additionally, SIRT6 expression levels in the spinal cord were estimated using immunoblotting with an anti-Flag antibody (Figure S3b). Furthermore, immunofluorescence determined the specific AAV-SIRT6 expression in spinal cord neuronal cells (Figure S3c). Functional behavioral evaluations were conducted at indicated time points (Figure 4a). Western blot results showed that AAV-SIRT6 mice exhibited higher GPX4 expression and decreased ACSL4 expression after SCI (Figure 4b). Furthermore, decreased 4-hydroxynonenal (4-HNE) expression, a well-established marker of lipid peroxidation, was observed in AAV-SIRT6 mice (Figure S3d). Behavioral assessments, including BMS scores, rotarod tests analysis, and footprint analysis indicated that AAV-SIRT6 injection triggered functional recovery comparative to the control vector AAV-Con (Figure 4c–e). To further corroborate these findings at the electrophysiological level, EMG recording of hindlimb muscles was performed. The results indicated that, compared to AAV-Con controls, AAV-SIRT6 treatment induced significantly greater MEPs in injured mice (Figure S3e). Consistently, DHE intensity was significantly decreased in the spinal cord of AAV-SIRT6 mice (Figure 4f). To identify anatomical correlates of functional recovery in mice, we assessed axon density at the lesion core of the injured spinal cord. As the main cytoskeleton protein of neuronal axons, neurofilament (NF) is highly expressed in neuronal axons and functions to maintain axonal structural stability and facilitate neuronal signal transduction. To evaluate axonal regeneration, we assessed neurofilament-positive (NF^+^) axons. While Wallerian degeneration typically results in more severe caudal axonal loss, our quantitative analysis focused on symmetrically defined regions adjacent to the lesion epicenter. At 28 days post-SCI, comparative to the Control, significantly more NF^+^ axons were detected within the lesion core of injured spinal cords in mice injected with AAV-SIRT6 (Figure 4g and h). Additionally, we employed the neuronal marker NeuN to stain surviving neurons in specific spinal cord regions (Z1–Z4) sited at defined distances from the lesion boundary in both groups. Analysis revealed that, compared to controls, the count of surviving NeuN^+^ neurons in regions Z1–Z3 was significantly elevated in the AAV-SIRT6 group (Figure 4i and j). Immunofluorescence also showed representative enlarged images of the same region in both groups (Figure 4k). These results indicate that SIRT6 overexpression in neuronal cells inhibits ferroptosis and triggers functional recovery after SCI *in vivo*.

### SIRT6 interacted with and deacetylated nuclear transcription factor erythroid 2-related factor 2 to alleviate ferroptosis

**Figure 5:**
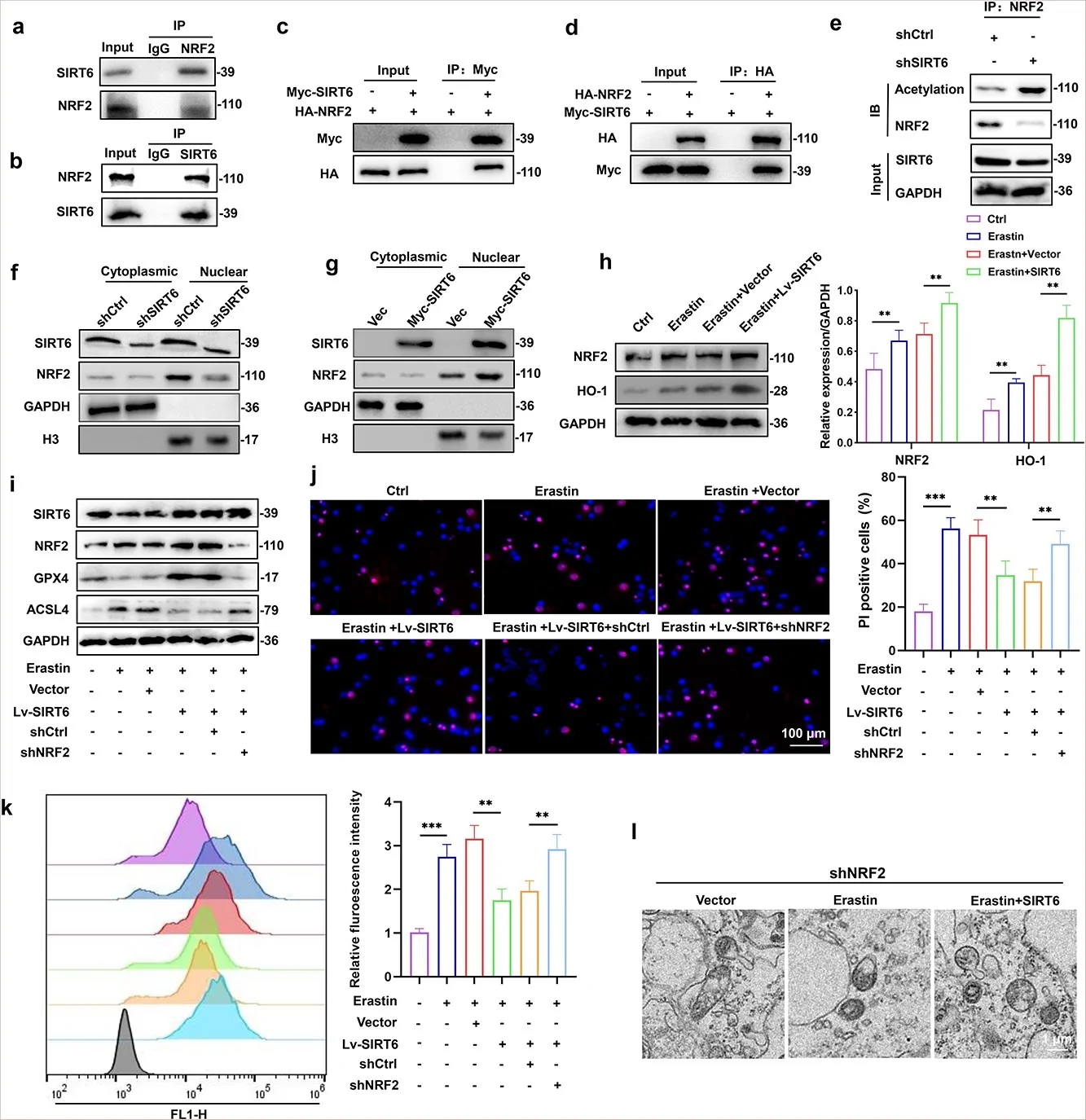
SIRT6 alleviates ferroptosis by up-regulating NRF2. (**a**-**b**) Detection of endogenous protein interactions between SIRT6 and NRF2 in neuronal cell lysates. (**c**-**d**) Detection of exogenous protein interactions between SIRT6 and NRF2 in HEK293T cells. Myc-tagged SIRT6 and HA-tagged NRF2 plasmids were transfected into HEK293T cells. (**e**) Deacetylation of NRF2 by SIRT6 in neurons. Acetylated NRF2 was analyzed by western blotting with anti-acetylation and total protein was evaluated in whole cell lysates (Input). (**f**-**g**) Relative levels of NRF2 in nuclear or cytoplasmic fractions were determined by subcellular fractionation analysis in neurons. (**h**-**l**) The neuronal cells were treated with 12µM Erastin for 24h, lentivirus containing SIRT6 (Lv-SIRT6) or control (vector) and control RNAi (shCtrl) or RNAi against NRF2 (shNRF2). (h) The expression levels of NRF2 and HO-1 were examined by western blot in vector or SIRT6 overexpressed neuronal cells with or without Erastin (left). Quantification of relative levels of NRF2 and HO-1 (right, *n* = 6). (i) Western blot analysis was used to detect the expression of ferroptosis-associated proteins (*n* = 6). (**j**) PI staining was used to detect cell survival in indicated groups (*n* = 6) (Scale bar: 100 μm). (**k**) ROS in indicated groups were measured (*n* = 6). (**l**) Representative morphology of mitochondria by TEM (Scale bar: 1 μm). ^*^p <0.05; ^*^^*^p <0.01; ^*^^*^^*^p <0.001. *SIRT6* Sirtuin 6, *NRF2* nuclear transcription factor erythroid 2-related factor 2, HO-1: heme oxygenase-1, *ACSL4* Acyl-CoA synthetase long chain family member 4, *GPX4* Glutathione peroxidase 4, *PI* propidium iodide, *ROS* reactive oxygen species, *TEM* transmission electron microscopy

We focused on the transcription factor NRF2, a critical player in redox biology, to investigate the mechanism of the neuroprotective role of SIRT6 in SCI ferroptosis. An earlier investigation showed that SIRT6 alleviates oxidative stress by interacting with and coactivating NRF2 in mesenchymal stem cells [22]. Therefore, we investigated whether SIRT6 binds to and activates NRF2 in neurons. Both exogenous and endogenous protein co-immunoprecipitation (Co-IP) showed the interaction between SIRT6 and NRF2 (Figure 5a–d). To deeply investigate whether SIRT6 deacetylates NRF2, we knocked down SIRT6 and detected the acetylation level of NRF2. The results illustrated that knockdown of SIRT6 led to an elevation in the acetylation level of NRF2 (Figure 5e). Next, Subcellular fractionation experiments further indicated that SIRT6 knockdown significantly reduces nuclear localization of NRF2 (Figure 5f and g). Western blot results demonstrated that NRF2 levels were significantly elevated in erastin-treated neuronal cells but elevated further in SIRT6 overexpressing cells (Figure 5h). The downstream target of NRF2, HO-1, exhibited the same pattern in response to erastin and/or SIRT6 (Figure 5h). Furthermore, we simultaneously overexpressed SIRT6 and downregulated NRF2 in neuronal cells. The western blotting illustrated that GPX4 expression was inhibited in shNRF2 cells, while ACSL4 expression was elevated compared with shNC cells (Figure 5i). SIRT6 overexpressing cells suppress erastin-induced ferroptosis, manifested by elevated cell viability, GSH levels, and decreased iron levels, ROS or lipid peroxidation as assessed by malondialdehyde, compared to erastin+vector cells (Figures 5j and k and S4a–d). These anti-ferroptosis effects of SIRT6 were significantly reduced in SIRT6 + shNRF2 cells (Figures 5j and k and S4a–d). Similarly, TEM studies showed that NRF2 knockdown abolishes the protective effect of SIRT6 on mitochondria (Figure 5l). These outcomes illustrate that SIRT6 mediates anti-ferroptosis effects by controlling NRF2 nuclear localization.

### STIP1 homology and U-box containing protein 1 interacts with **SIRT6**

**Figure 6:**
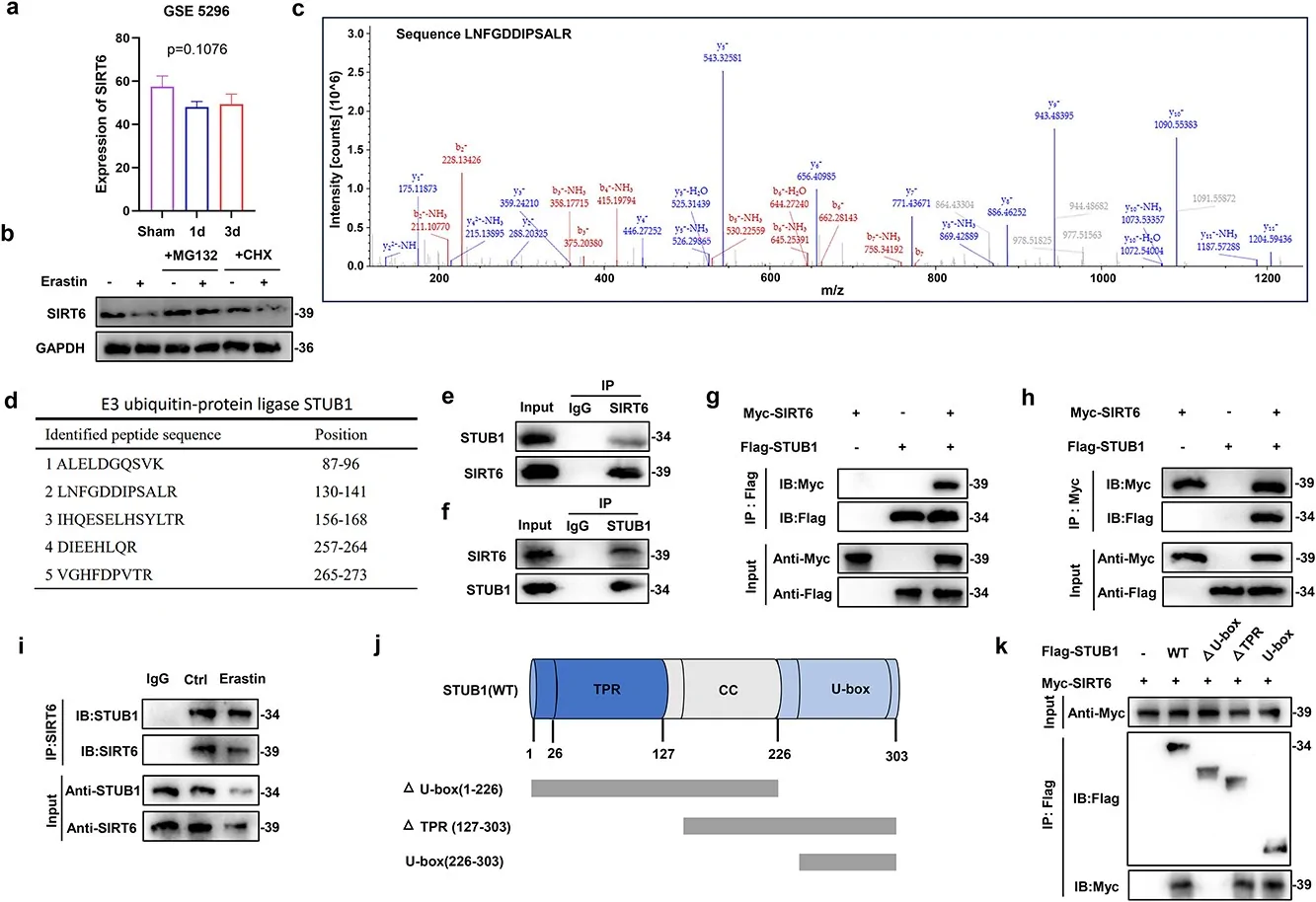
STUB1 interacts with SIRT6. (**a**) The expression of SIRT6 in SCI tissues from GSE5296. (**b**) Western blot for SIRT6 in neurons treated with/without Erastin for 48 h, with/without proteasome inhibitor MG132 (1 µmol/L) or cycloheximide (CHX, 10 μg/ml). (**c**) Representative IP/MS spectrum of an identified STUB1 peptide. (**d**) Information for the MS-identified peptides. (**e**-**f**) Detection of endogenous protein interactions between STUB1 and SIRT6 in neuronal cell lysate. (**g**-**h**) Detection of exogenous protein interactions between STUB1 and SIRT6 in HEK293T cells. Flag-tagged STUB1 and Myc-tagged SIRT6 plasmids were transfected into HEK293T cells. (**i**) Immunoprecipitation for SIRT6 with neurons treated with erastin for 48 h, followed by western blot for STUB1 and SIRT6. IgG used as isotype control. (**j**) Schematic representations of Flag-tagged full-length (FL) STUB1, and various deletion mutants. (**k**) Myc-SIRT6 and Flag-tagged deletion mutations of STUB1 were co-transfected into HEK293T cells, and cell lysates were analyzed by immunoprecipitation followed by immunoblotting with anti-Myc and anti-Flag. ^*^p <0.05; ^*^^*^p <0.01; ^*^^*^^*^p <0.001. *SIRT6* Sirtuin 6, *STUB1* STIP1 homology and U-box containing protein 1

*In vivo* and *in vitro* trials have indicated that SIRT6 was decreased throughout ferroptosis. Interestingly, the SIRT6 transcription level is inconsistent with its translation. As mentioned above, we discovered that SIRT6 mRNA expression decreased slightly and lagged protein levels in SCI (Figure 1a and i). SCI-related dataset (GEO: GSE5296) analysis revealed that the change of SIRT6 mRNA after SCI was not significantly different (Figure 6a). Therefore, we hypothesize that post-translational modifications may be involved in regulating SIRT6 stability. SIRT6 expression was increased by proteasome inhibitor MG132 treatment but not by the protein synthesis inhibitor cycloheximide (CHX) treatment (Figure 6b), indicating that SIRT6 expression is mainly determined by protein stability and proteasomal degradation. We used IP/MS to investigate which proteins interact with SIRT6 to identify the mechanism by which it decreases during ferroptosis. IP/MS identified STUB1 as a protein that may interact with SIRT6 (Figure 6c and d), which is involved in neuronal oxidative stress regulation [26, 27]. We validated this interaction in neuronal cells using Co-IP. STUB1 specifically immunoprecipitated SIRT6, and reciprocal Co-IP confirmed that SIRT6 also robustly immunoprecipitated STUB1 (Figure 6e and f). To further corroborate this finding, we performed Co-IP in HEK293T cells expressing epitope-tagged proteins. Flag-tagged STUB1 co-precipitated with Myc-tagged SIRT6, and conversely, Myc-tagged SIRT6 co-precipitated with Flag-tagged STUB1 (Figure 6g and h). Notably, this interaction was attenuated following erastin treatment (Figure 6i). STUB1 contains several functional domains: a TPR domain crucial for chaperone binding, a coiled-coil (CC) domain, and a U-box domain accountable for its ubiquitin ligase activity. To map the SIRT6-binding region on STUB1, we constructed a series of truncated FLAG-tagged STUB1 variants (Figure 6j). Co-IP analysis revealed that both the isolated U-box domain and the ΔTPR deletion mutant interacted with SIRT6, indicating that the U-box domain facilitates the physical interaction between STUB1 and SIRT6 (Figure 6k). Collectively, these results demonstrate a direct physical interaction between STUB1 and SIRT6.

### STIP1 homology and U-box containing protein 1 positively regulates SIRT6 stability and promotes K63-linked ubiquitination of SIRT6

**Figure 7:**
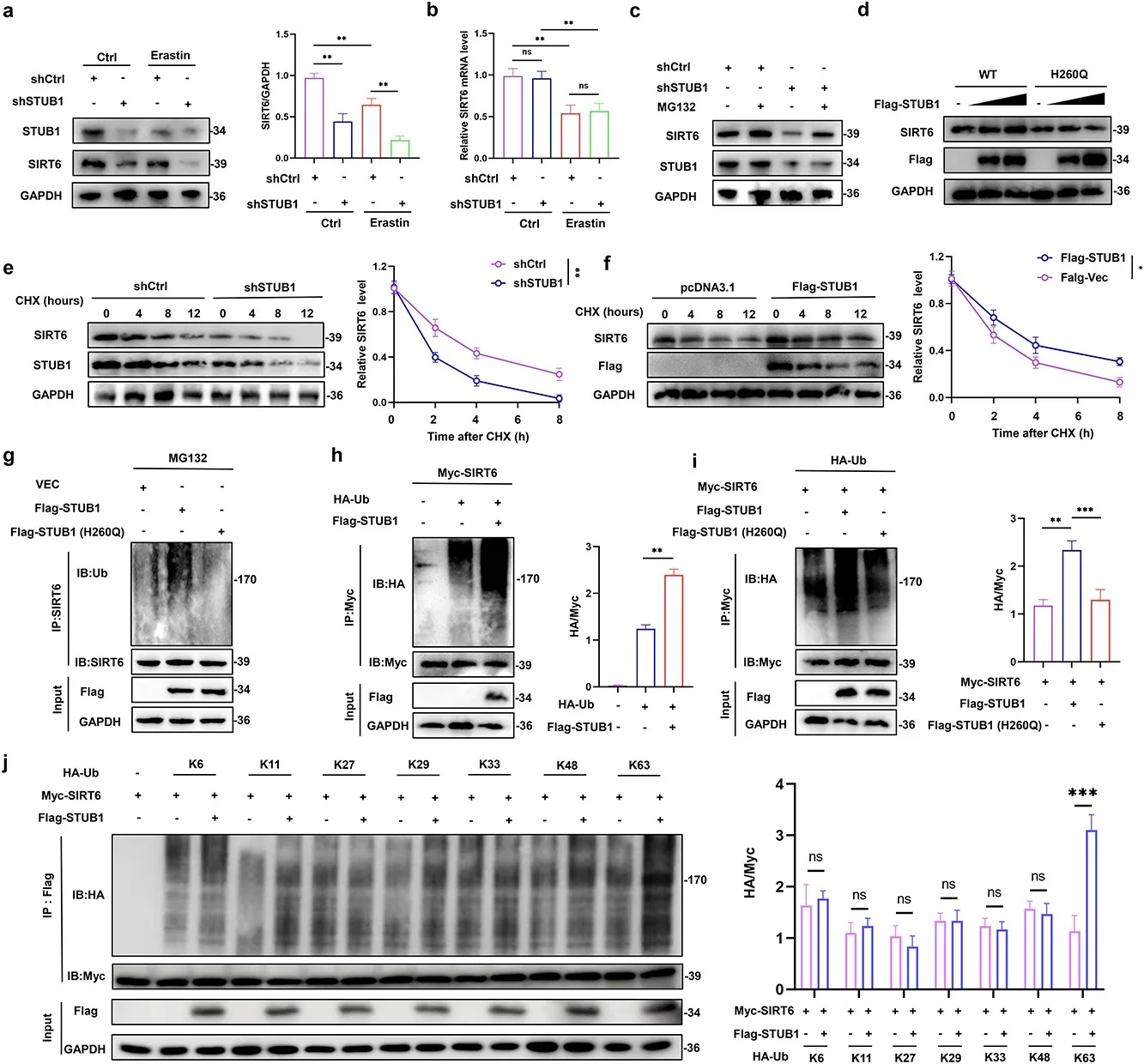
STUB1 positively regulates SIRT6 stability and promotes K63-linked ubiquitination of SIRT6. (**a**) Representative immunoblots (left) and quantification of protein level of SIRT6 (right, *n* = 3). (**b**) mRNA expression was analyzed by qPCR under Erastin treatment (*n* = 3). (**c**) Evaluation of SIRT6 protein levels in neurons transfected with Lv-shSTUB1 with or without proteasome inhibitor MG132 treatment. (**d**) Increasing amounts of Flag-tagged STUB1 (WT or H260Q mutant) were transfected and the expression levels of SIRT6 and Flag-tagged STUB1 were detected by western blot (*n* = 3). (**e**) Detection of SIRT6 protein levels in shNC and shSTUB1 transfected neuronal cells in the presence of CHX (10 μg/ml) for indicated time point (*n* = 3). (**f**) Detection of SIRT6 protein levels in neuronal cells with overexpression of STUB1 or corresponding control in the presence of CHX (10 μg/ml) for the indicated time points (*n* = 3). (**g**) Primary neurons were transfected with Flag-STUB1 (WT or H260Q mutant) or vector control. Cell lysates were immunoprecipitated with anti-SIRT6 and immunoblotted with the indicated antibodies in the presence of MG132 (*n* = 3). (**h**) Lysates from HEK 293 T cells transfected with HA-tagged Ub and Myc-tagged SIRT6 together with Flag-tagged STUB1 were immunoprecipitated with anti-Myc, followed by immunoblotting with anti-HA and anti-Myc (left). Quantification of relative Ub-SIRT6 levels (right, *n* = 3). (**i**) Lysates from HEK293T cells transfected with HA-tagged Ub and Myc-tagged SIRT6 together with Flag-tagged STUB1 (WT) or Flag-tagged STUB1 (H260Q) were immunoprecipitated with anti-Myc, followed by immunoblotting with anti-HA and anti-Myc (left). Quantification of relative Ub-SIRT6 levels (right, *n* = 3). (**j**) HEK293T cells were co-transfected with Myc-SIRT6, Flag-STUB1, and HA-Ub plasmids containing different lysine residues (K6, K11, K27, K29, K33, K48, or K63), followed by analysis of SIRT6 ubiquitination linkage (*n* = 3). ^*^p <0.05; ^*^^*^p <0.01; ^*^^*^^*^p <0.001. *SIRT6* Sirtuin 6, *STUB1* STIP1 homology and U-box containing protein 1

As STUB1 interacts with SIRT6, the impact of silencing STUB1 on SIRT6 levels in neurons was examined. STUB1 knockdown reduced SIRT6 protein expression level compared to shCtrl (Figure 7a). Erastin treatment significantly decreased STUB1 and SIRT6 protein expressions in control shRNA-treated cells comparative to the control and suppressed SIRT6 further in STUB1-depleted cells (Figure 7a). Notably, STUB1 silencing or overexpression had no influence on SIRT6 mRNA level with or without the erastin treatment (Figure 7b). This suggests that SIRT6 regulation by STUB1 is not at the mRNA level, but rather at the level of SIRT6 protein stability (Figure 7b). Furthermore, the proteasome inhibitor MG132 overexpressed endogenous SIRT6 in shSTUB1 neuronal cells, indicating that STUB1 prevents proteasome-dependent degradation of SIRT6 (Figure 7c). These results demonstrate that STUB1 depletion increases proteasome-mediated degradation of SIRT6. Moreover, it was discovered that STUB1 overexpression elevated the SIRT6 protein level, while upregulation of the catalytically inactive H260Q mutant STUB1 abolished this effect (Figure 7d). We then investigated the effect of STUB1 deletion on SIRT6 protein stability in the presence of the protein synthesis inhibitor CHX. STUB1 silencing accelerated SIRT6 degradation and shortened its half-life compared to control cells, while STUB1 overexpression significantly inhibited SIRT6 degradation, confirming that STUB1 regulates the stability of SIRT6 protein (Figure 7e and f). Since STUB1 is an E3 ubiquitin ligase that controls SIRT6 stability through its ubiquitin ligase activity, we examined whether STUB1 controls SIRT6 ubiquitination. Notably, ectopically overexpressed STUB1 significantly promoted ubiquitination of SIRT6 in primary neurons, while H260Q STUB1 did not (Figure 7g). Conversely, suppression of STUB1 decreased ubiquitination of endogenous SIRT6 (Figure S5a). Next, co-transfection of HEK293T cells with Myc-SIRT6, Flag-STUB1 (WT or H260Q mutant), and HA-Ub was conducted. WT STUB1 overexpression promotes SIRT6 ubiquitination (Figure 7h), an impact that can be reversed by the H260Q mutant STUB1 (Figure 7i). Polyubiquitin chains can be formed through various linkage types, such as K6/11/27/29/33/48/63. To determine the specific polyubiquitin linkage type on SIRT6 regulated by STUB1, we transfected HEK293T cells with different ubiquitin mutants and performed ubiquitination assays. The results demonstrated that STUB1 significantly promotes K63-linked ubiquitination of SIRT6, while other linkage types showed no significant changes (Figure 7j). Collectively, these findings establish that STUB1 enhances SIRT6 protein stability by mediating K63-linked polyubiquitin chains.

### STIP1 homology and U-box containing protein 1 suppresses ferroptosis and triggers functional recovery by stabilizing SIRT6 *in vivo*

**Figure 8:**
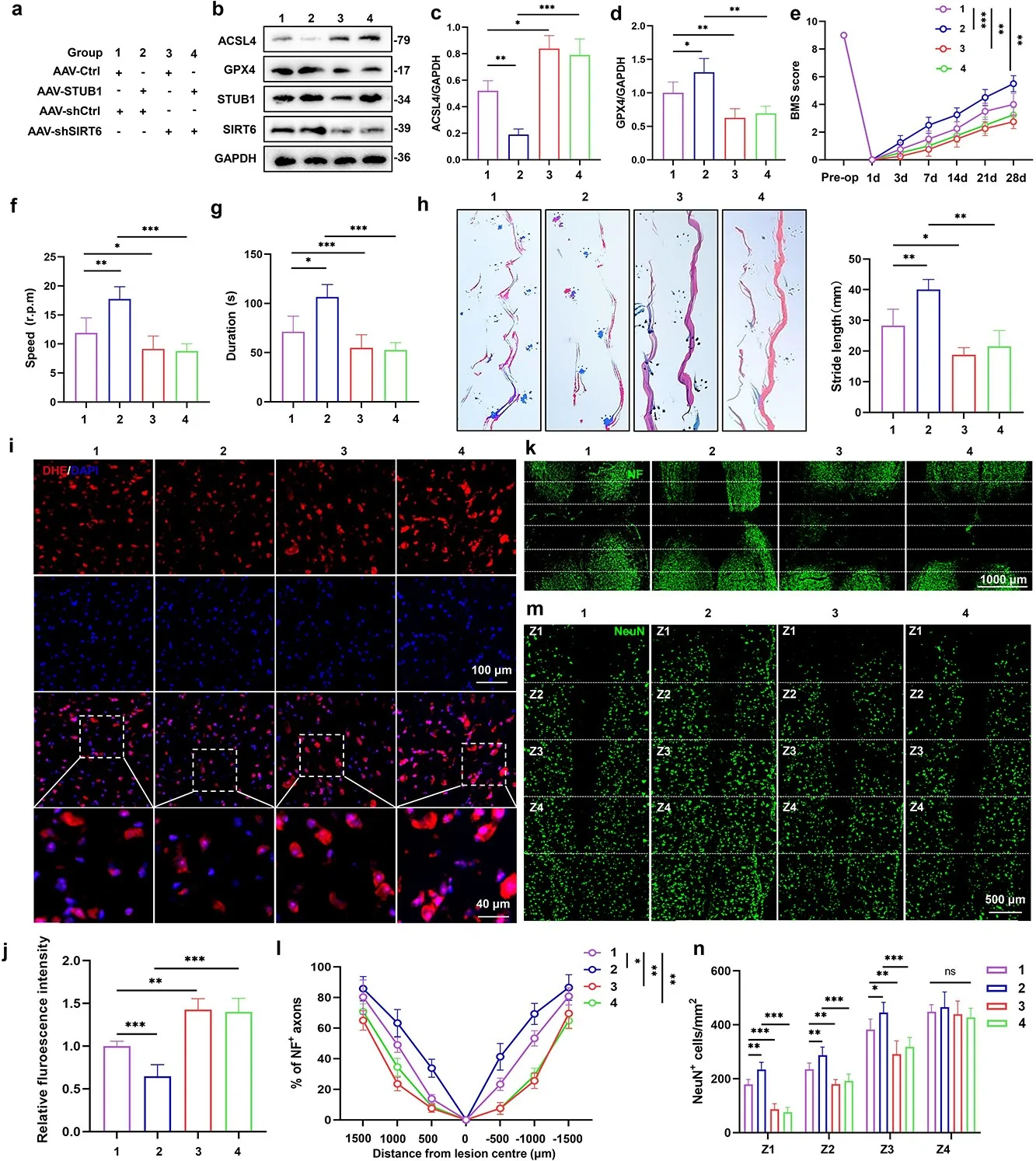
STUB1 inhibits ferroptosis and promotes functional recovery by stabilizing SIRT6 *in vivo*. (**a**) Detailed information of test groups. (**b**-**d**) Representative immunoblots showing expression levels of ferroptosis-associate proteins in injured spinal cord at 7 days postinjury (left). Quantification of relative levels of relatived protein (right, *n* = 6). (**e**) BMS score results after SCI (*n* = 6). (**f**-**h**) Rotarod test and footprint results at 28 days postinjury (*n* = 6). (**i**-**j**) DHE staining was used to detect ROS levels in the injured spinal cord at 7 days postinjury (*n* = 6) (Scale bar: 100 μm; 40 μm). (**k**-**l**) Representative immunofluorescence images for NF in injured spinal cords at 28 days postinjury (*n* = 3) (Scale bar: 1000 μm) and quantitative analysis of NF positive area. (**m**-**n**) Representative immunofluorescence images of NeuN^+^ neurons in Z1-Z4 regions adjacent to lesion core at 28 days postinjury (*n* = 3) (Scale bar: 500 μm) and quantification of NeuN^+^ neurons. ^*^p <0.05; ^*^^*^p <0.01; ^*^^*^^*^p <0.001. *SIRT6* Sirtuin 6, *ACSL4* Acyl-CoA synthetase long chain family member 4, *GPX4* Glutathione peroxidase 4, *STUB1* STIP1 homology and U-box containing protein 1, *BMS* basso mouse scale, *SCI* spinal cord injury, *DHE* dihydroethidium, *ROS* reactive oxygen species, *NF200* neurofilament 200, *NeuN* neuronal nuclei

After demonstrating that STUB1 binds and stabilizes SIRT6, we investigated whether STUB1 inhibits ferroptosis and triggers functional recovery through SIRT6. We generated neuronal cell-specific STUB1 upregulation and SIRT6 suppression mice models by injecting AAV-STUB1 and AAV-shSIRT6, respectively (Figure 8a). These results suggest that STUB1 overexpression inhibits ferroptosis and promotes functional recovery, and increases the count of surviving neurons after SCI compared to AAV-Con (Figures 8b–n and S6a and b). Importantly, these protective effects were markedly attenuated in mice co-injected with AAV-shSIRT6 (Figures 8b–n and S6a and b). Collectively, these findings underline the vital function of the STUB1-SIRT6 axis in controlling ferroptosis and functional recovery after SCI.

### STIP1 homology and U-box containing protein 1-stabilized SIRT6 results in increased nuclear transcription factor erythroid 2-related factor 2 activity and nuclear transcription factor erythroid 2-related factor 2–knockout abolishes the neuroprotective effects of STIP1 homology and U-box containing protein 1/SIRT6 activation in SCI

**Figure 9:**
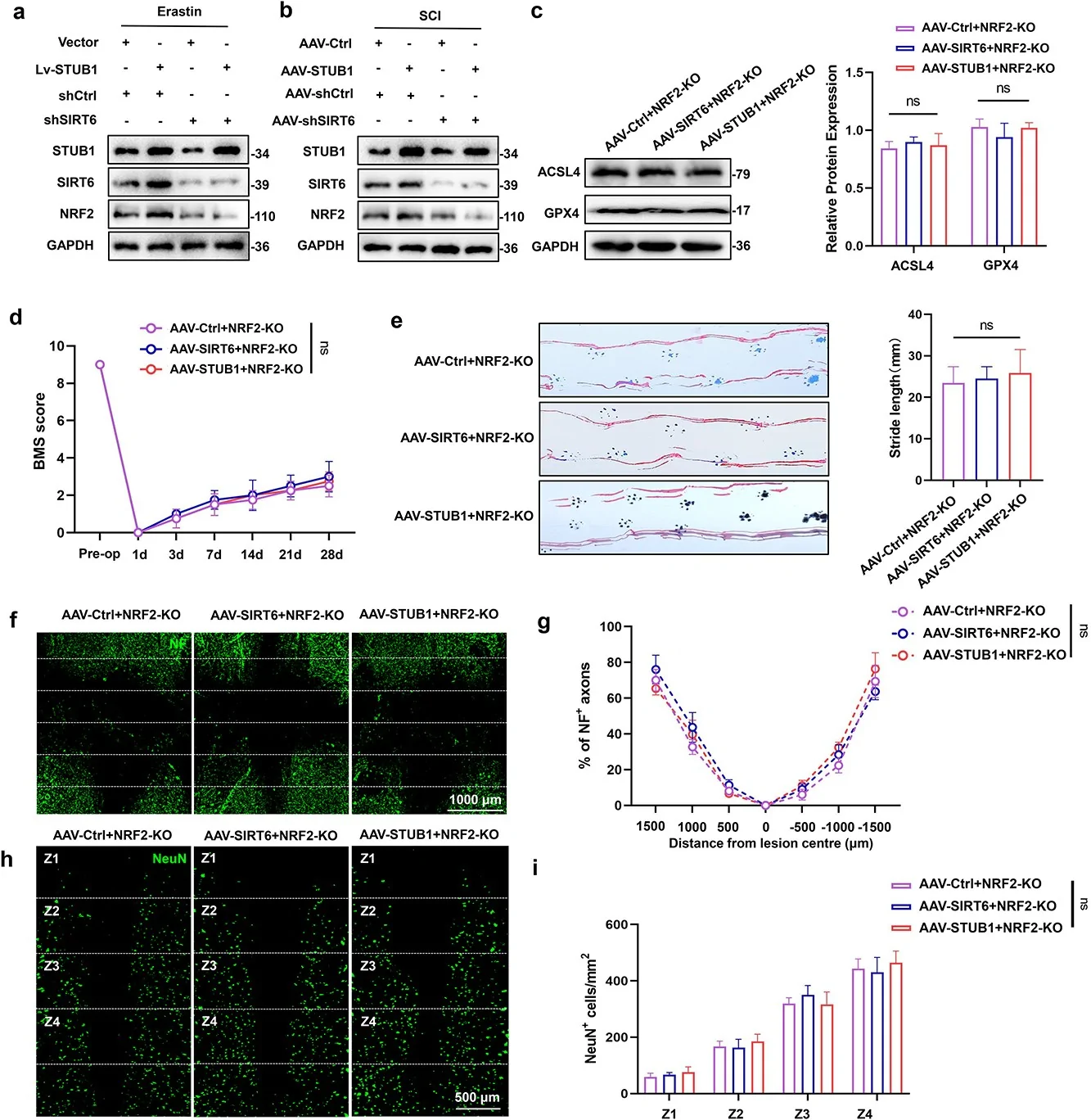
NRF2 knockout abolishes the neuroprotective effects of STUB1/SIRT6 activation in SCI. (**a**) Representative western blot showing the expression of NRF2 proteins in neuronal cells (*n* = 3). (**b**) Representative western blot showing the expression of NRF2 proteins in the injured spinal cord (*n* = 3). (**c**) Protein expression levels of ferroptosis-associated proteins detected by western blot at day 7 post-injury (left). Quantification of relative levels of ferroptosis-associated proteins (right, *n* = 6). (**d**) BMS score results after SCI (*n* = 6). (**e**) Footprint results on day 28 after SCI (*n* = 6). (**f**) Representative immunofluorescence images for NF in injured spinal cords at 28 days postinjury (*n* = 3) (Scale bar: 1000 μm). (**g**) Quantitative analysis of NF positive area. (**h**) Representative immunofluorescence images of NeuN^+^ neurons in Z1-Z4 regions adjacent to lesion core at 28 days postinjury (*n* = 3) (Scale bar: 500 μm). (**i**) Quantification of NeuN^+^ neurons in Z1-Z4 regions adjacent to lesion core. ^*^p <0.05; ^*^^*^p <0.01; ^*^^*^^*^p <0.001. *SIRT6* Sirtuin 6, *ACSL4* Acyl-CoA synthetase long chain family member 4, *GPX4* glutathione peroxidase 4, *STUB1* STIP1 homology and U-box containing protein 1, NRF2: Nuclear transcription factor erythroid 2-related factor 2, *BMS* basso mouse scale, *SCI* spinal cord injury, *NF200* neurofilament 200, *NeuN* neuronal nuclei

We further investigated whether STUB1 promotes NRF2 activity. Endogenous STUB1 overexpression significantly elevated the mRNA levels of numerous known NRF2 transcription targets (e.g. HO-1, GCLC, GCLM, and NQO1) (Figure S7a). To further demonstrate that STUB1 promotes NRF2 activity through SIRT6, we investigated whether SIRT6 knockdown reverses this STUB1 stimulative activity. The stimulative activity of STUB1 to NRF2 transcriptional activity was reversed by SIRT6 knockdown (Figure S7a). Western blotting illustrated that SIRT6 suppression abolished the stimulatory effect of STUB1 overexpression on NRF2 *in vitro* and *in vivo* (Figure 9a and b). As demonstrated in Figures 4 and 8, STUB1 or SIRT6 overexpression in wild-type (WT) mice significantly inhibited ferroptosis and enhanced functional recovery post-SCI. To determine whether these protective effects require NRF2, we performed parallel experiments in NRF2-KO mice. We administered AAV-Con and AAV-STUB1 or SIRT6 to NRF2 KO mice post-SCI to clarify the function of NRF2 in STUB1/SIRT6-mediated neuroprotection. Western blot analysis revealed no differences in ferroptosis-related protein changes in NRF2 KO mice (Figure 9c). Furthermore, both BMS scores and footprint analysis revealed no significant improvement in functional recovery (Figure 9d and e). Furthermore, no variation in the count of surviving neurons among the three groups was observed (Figure 9f–i). These results suggest that STUB1/SIRT6 promotes functional recovery and inhibits ferroptosis via NRF2 *in vivo*.

## Discussion

Herein, we demonstrate that ferroptosis contributes to the pathological progression of SCI, and that suppression of ferroptosis promotes functional recovery in mice. Through several loss- and gain-of-function experiments both *in vitro* and *in vivo*, we establish that SIRT6 has a critical function in inhibiting neuronal ferroptosis. Mechanistically, SIRT6 deacetylates NRF2, promoting its nuclear translocation and subsequent stimulation of AREs. The anti-ferroptotic effect of the SIRT6/NRF2 axis was confirmed by rescue experiments. Furthermore, we determine the E3 ubiquitin ligase STUB1 as an upstream regulator that stabilizes SIRT6 through K63-linked ubiquitination, thereby enhancing its protein stability. Collectively, our outcomes illustrate that the STUB1/SIRT6/NRF2 axis is a prospective therapeutic target for SCI.

SCI is a devastating neurological disorder with limited treatments. While surgical stabilization remains the standard of care to prevent secondary mechanical damage, effective pharmacological interventions that inhibit secondary cell death are still lacking [28–32]. Thus, the mechanism of SCI onset should be extensively examined to establish successful therapeutic strategies. Ferroptosis has been reported to have pathogenic effects on major neurological disorders (e.g. traumatic brain injury, Parkinson’s disease, stroke, and Alzheimer’s disease) [33–36]. Recent investigations have illustrated that ferroptosis, as a neuronal death mechanism, mediates the occurrence of SCI. Local iron overload and ROS activation after SCI increase the excitotoxicity of glutamate, an important factor in the severity of secondary injury [37]. Several regulators of ferroptosis have been identified, such as ALOX12, YAP1, FSP1, and USP11, which control iron metabolism and lipid peroxidation [9, 38–41]. Nonetheless, the upstream regulatory networks governing ferroptosis after SCI remain poorly understood.

Sirtuin family members have been implicated in various neurodegenerative disorders [42–45]. However, research on sirtuin functions in SCI remains limited. SIRT6, a nuclear deacetylase targeting histone H3K9 and H3K56, regulates lifespan and modulates transcription factors including NF-κB, HIF-1α and c-JUN [46–48].It is noteworthy that SIRT6 silencing could overcome resistance to sorafenib-triggered ferroptosis in gastric cancer cells [49]. Given the distinct role of SIRT6 in each of these processes and the vital function of ferroptosis in SCI, we examined the specific function of neuronal SIRT6 in post-SCI pathophysiology using animal models.

We demonstrate that SIRT6 functions as an anti-ferroptosis protein to promote functional recovery after SCI, positioning it as a promising therapeutic target. Supporting evidence shows that SIRT6 expression is downregulated following SCI and during erastin-induced ferroptosis, while its overexpression reduces neuronal cytotoxicity and lipid peroxidation *in vitro*, and suppresses ferroptosis to improve prognosis *in vivo*. We further demonstrated that this neuroprotective effect could be reversed through NRF2 downregulation, a critical transcription regulator of redox homeostasis and ferroptosis [50]. NRF2 stimulates antioxidant genes such as HO-1 by binding to AREs [51]. NRF2 expression is tightly regulated at multiple levels and maintained at low levels under physiological conditions. As a transcription factor, NRF2 regulates several genes representing the ferroptosis cascade (e.g. GPX4, SLC7A11, ferroportin, and ferritin) [52–54]. Therefore, targeting NRF2 to reverse ferroptosis is valuable for certain disorders (e.g. neurodegenerative diseases and cancer) [55, 56]. Numerous studies have shown that SIRT6 can stimulate NRF2 expression to mediate antioxidant effects in different disease models, including cerebral ischemia–reperfusion, acute liver failure, and acute kidney injury [57–59]. Nonetheless, the regulatory effects of the SIRT6-NRF2 axis in SCI-related oxidative stress remain unknown. Of note, the negative correlation between NRF2 activity and SCI onset has been reported [60]. We discovered that SIRT6 upregulation stimulated NRF2 and its target gene, HO-1 and decreased oxidative stress. Furthermore, the neuroprotective effects of SIRT6 upregulation *in vitro* were abolished upon NRF2 suppression. Notably, while SIRT6 stimulates the NRF2 pathway, its expression is downregulated following SCI. This compromised SIRT6-activated, NRF2-dependent antioxidant and cellular defense response thus potentially contributes to neuronal ferroptosis after SCI. Taken together, these outcomes consider NRF2 as a vital downstream mediator of SIRT6’s neuroprotective function in the context of SCI.

To examine the initial mechanism of neuronal SIRT6 changes, we assessed SIRT6 mRNA levels *in vivo* in response to SCI. Despite the decrease in the SIRT6 protein level, the decreased mRNA level of SIRT6 significantly lagged behind the protein level. Furthermore, SCI-related dataset analysis revealed that the change in SIRT6 mRNA did not reach a statistical difference after SCI. Similar results were observed in primary neurons exposed to erastin-induced ferroptosis, suggesting that post-translational modifications may significantly regulate SIRT6 stability and activation.

To clarify the mechanism of SIRT6 depletion throughout ferroptosis further, we conducted IP/MS and detected STUB1 protein as a probable SIRT6-interacting protein in neuronal cells. STUB1 is an E3 ligase that degrades misfolded substrates through the proteasome pathway and participates in numerous physiological and pathological processes, including protein quality control, cell aging, immunity, and neurological disease [61–63]. STUB1 possesses distinct functional domains: an N-terminal TPR domain comprising three tetratricopeptide repeats, which mediates interactions with chaperone proteins (Hsc70, Hsp70, and Hsp90), and a C-terminal U-box domain, which recruits ubiquitin-conjugating enzymes (E2s) and confers its E3 ubiquitin ligase activity [64]. STUB1 controls various disease processes through the ubiquitin-proteasome system [65]. A recent study has found a link between STUB1 and ferroptosis by demonstrating that STUB1 promotes IRP2 ubiquitination degradation and thus inhibits ferroptosis in liver fibrosis [66]. Furthermore, STUB1 is essential for regulating mitochondrial integrity and neuronal survival after oxygen and glucose deprivation; loss of STUB1 renders neurons unable to mount a protective response after stress and more vulnerable to an acute injury [26, 27].

Here, we demonstrate that SIRT6 physically interacts with STUB1 in both neurons and the injured spinal cord. Importantly, STUB1, functioning as an E3 ubiquitin ligase, positively controls SIRT6 protein stability in these contexts. Further *in vitro* experiments showed that STUB1 overexpression protects SIRT6 from proteasomal degradation, whereas STUB1 knockdown accelerates its ubiquitin-mediated degradation. Of note, STUB1 silencing or overexpression in neurons had no effect on SIRT6 mRNA expression. It is well-established that polyubiquitin chains are primarily assembled through linkages at Lys48 (K48) or Lys63 (K63) residues. Among these, K48-linked polyubiquitin chains serve as the canonical targeting signal for proteasomal degradation of substrate proteins. Previous studies have reported that STUB1 normally destabilizes substrate proteins through K48-linked ubiquitination. However, our study shows that STUB1 increases SIRT6 ubiquitination and stability. This suggests that STUB1 may indirectly stabilize SIRT6 by degrading its negative regulators or stabilize SIRT6 by noncanonical ubiquitination rather than canonical K48-linked ubiquitination. In fact, the IP/MS results identified several other E3 ligases that may interact with SIRT6. It will be interesting to investigate whether STUB1 and other E3 ligases can antagonize each other and balance the levels of SIRT6 and other correlated proteins. Future research will concentrate on determining other factors that contribute to SIRT6 stability and their probable control by STUB1.

This study presents several key innovations in comprehending the molecular mechanisms underlying ferroptosis after SCI. We are the first to identify SIRT6 as a vital negative controller of ferroptosis in this context, thereby expanding the current understanding of Sirtuin family functions in central nervous system pathology. Furthermore, we uncover a non-canonical regulatory mechanism wherein the E3 ubiquitin ligase STUB1 stabilizes SIRT6 via K63-linked ubiquitination, which contrasts with the conventional K48-linked degradative ubiquitination typically mediated by STUB1; this finding highlights its context-dependent dual role in protein stabilization. In addition, we delineate a complete STUB1/SIRT6/NRF2 signaling axis that integrates post-translational modification, nuclear translocation, and transcriptional activation, providing a novel mechanistic framework linking protein stability to antioxidant defense and ferroptosis inhibition. Utilizing gain- and loss-of-function strategies *in vitro* and *in vivo*, along with NRF2-KO mice, we provide robust genetic and pharmacological evidence that this axis is essential for neuroprotection after SCI, offering a promising therapeutic target for intervention. Despite these advances, several limitations should be acknowledged. First, ferroptosis is only one of multiple cell death pathways activated after SCI; apoptosis, pyroptosis, and autophagic cell death also contribute to neuronal loss, and their relative contributions were not systematically compared in this study. Our *in vitro* model primarily employed erastin to induce ferroptosis, whereas the post-SCI microenvironment involves complex ischemic, hypoxic, and inflammatory stimuli that may not be fully recapitulated by a single chemical inducer. Although we supplemented our functional assessments with EMG analysis to evaluate neuromuscular function, this study did not directly measure action potential conduction velocity across the lesion site or synaptic transmission efficiency. Future investigations employing direct MEP recordings and electrophysiological analyses at the synaptic level will be necessary to comprehensively validate the functional benefits of the STUB1/SIRT6/NRF2 axis. Moreover, while we identified STUB1 as a key regulator of SIRT6, whether it modulates ferroptosis through additional substrates remains unknown, as our IP/MS data revealed other potential STUB1-interacting proteins that warrant further investigation.

## Conclusions

In conclusion, ferroptosis is strongly associated with SCI. Understanding the molecular mechanisms and signaling pathways governing ferroptosis may present innovative diagnostic and therapeutic strategies for modulating neuronal survival and death post-SCI. We demonstrate a critical role for SIRT6 in protecting neurons from ferroptosis and NRF2 mediated anti-ferroptosis effect. Furthermore, we reveal that STUB1-mediated post-translational regulation of SIRT6 stability is essential for this protective function in neuronal cells. Collectively, these findings identify the STUB1/SIRT6/NRF2 axis as a promising therapeutic target for SCI treatment.

## Abbreviations

SCI: spinal cord injury; PCD: programmed cell deaths; SCIRI: spinal cord ischemia–reperfusion injury; GPX4: glutathione peroxidase 4; NRF2: nuclear transcription factor erythroid 2-related factor 2; ARE: antioxidant response elements; NAD: nicotinamide adenine dinucleotide; RUNX2: runt-related transcription factor 2; SIRT6: sirtuin 6; STUB1: STIP1 homology and U-box containing protein 1; BMS: Basso mouse scale; 4-HNE: 4-hydroxynonenal; EMG: electromyography; MEPs: motor evoked potentials; BSA: bovine serum albumin; GFAP: glial fibrillary acidic protein; NeuN: neuronal nuclei; Iba1: ionized calcium-binding adapter molecule 1; NF200: neurofilament 200; DAPI: 4′,6-diamidino-2-phenylindole; DMEM: Dulbecco’s modified eagle medium; RNA: ribonucleic acid; GAPDH: glyceraldehyde-3-phosphate dehydrogenase; MOI: multiplicity of infection; PI: propidium iodide; MDA: malondialdehyde; GSH: glutathione; ROS: reactive oxygen species; DHE: dihydroethidium; TEM: transmission electron microscopy; ACSL4: acyl-CoA synthetase long chain family member 4; CD31: cluster of differentiation 31; Fer-1: ferrostatin-1; IP/MS: immunoprecipitation coupled with mass spectrometry; HO-1: heme oxygenase-1; GCLC: glutamate-cysteine ligase catalytic subunit; GCLM: glutamate-cysteine ligase modifier subunit; NQO1: NADPH quinone dehydrogenase 1; ALOX12: arachidonate 12-lipoxygenase; YAP1: Yes1 associated transcriptional regulator; FSP1: ferroptosis suppressor protein 1; USP11: ubiquitin specific peptidase 11; NF-κB: nuclear factor kappa-B; HIF-1α: hypoxia-inducible factor 1-alpha; SLC7A11: solute carrier family 7 member 11; IRP2: iron regulatory protein 2.

## Supplementary Material

Supplementary_material_tkag047

## Data Availability

Available upon reasonable request.
