# Supplementary material for: STUB1 stabilizes sirtuin 6 via K63-linked ubiquitination to promote NRF2 deacetylation for enhanced ferroptosis resistance after spinal cord injury
Source: Burns Trauma. 2026 Jul 14;14:tkag047. doi: 10.1093/burnst/tkag047 (PMC13580083; doi:10.1093/burnst/tkag047)
Supplement: Supplementary file 1 [file supplementary_material_tkag047.zip › Figure S1.docx]

**Figure S1**

**Figure S1:Fer-1 promotes functional recovery after SCI.**

**a** Representative image of footprint analysis at day 28 post-injury (blue, front paw print; red, hind paw print). **b-c** Rotarod tests at day 28 post-injury (n = 6). **d** Quantitative analysis of SIRT6 co-localization with different cell type-specific markers(n = 3). **e** Quantification of SIRT6 expression(n = 3). *p <.05; **p <.01; ***p <.001. SCI: Spinal cord injury; SIRT6:Sirtuin 6; GFAP :Glial fibrillary acidic protein; NeuN: Neuronal nuclei; Iba1:Ionized calcium-binding adapter molecule 1; CD31:Cluster of differentiation 31.
