# Supplementary material for: STUB1 stabilizes sirtuin 6 via K63-linked ubiquitination to promote NRF2 deacetylation for enhanced ferroptosis resistance after spinal cord injury
Source: Burns Trauma. 2026 Jul 14;14:tkag047. doi: 10.1093/burnst/tkag047 (PMC13580083; doi:10.1093/burnst/tkag047)
Supplement: Supplementary file 1 [file supplementary_material_tkag047.zip › Figure S2.docx]

**Figure S2**

 **Figure S2:** **Fer-1 treatment reduces cell death and reverses ferroptosis events.**

**a** CCK-8 assay of neurons with Fer-1 or the equal volume of DMSO. **b-e** The relative values of Fe^2+^ concentrations, MDA, GSH and ROS in indicated groups were measured (n = 6). *p <.05; **p <.01; ***p <.001. MDA: Malondialdehyde; GSH: Glutathione; ROS: Reactive oxygen species.
