# Supplementary material for: STUB1 stabilizes sirtuin 6 via K63-linked ubiquitination to promote NRF2 deacetylation for enhanced ferroptosis resistance after spinal cord injury
Source: Burns Trauma. 2026 Jul 14;14:tkag047. doi: 10.1093/burnst/tkag047 (PMC13580083; doi:10.1093/burnst/tkag047)
Supplement: Supplementary file 1 [file supplementary_material_tkag047.zip › Figure S3.docx]

**Figure S3**

 **Figure S3: Transfection efficiency of AAV-PHP.eB-hSyn-SIRT6** **and its effects on ferroptosis and electrophysiological function in mice.**

**a** Representative immunoblots (left) and quantification of SIRT6 in spinal cord (right). **b** Representative immunoblots of Flag (SIRT6) protein level in spinal cord. **c** Representative image of immunofluorescence to identify transfection and neuronal cell specificity of AAV-SIRT6. (Scale bar = 100 μm).**d** Representative western blot showing the expression of 4HNE protein in the injured spinal cord at 7 days postinjury (left). Quantification of relative levels of related protein (right, n = 6).**e** Representative images and quantification of EMG recordings of gastrocnemius muscle in mice at 28 days postinjury (n = 6). *p <.05; **p <.01; ***p <.001. SCI: Spinal cord injury;SIRT6:Sirtuin 6; GAPDH: Glyceraldehyde-3-phosphate Dehydrogenase; NeuN: Neuronal nuclei; 4-HNE:4-hydroxynonenal; EMG: Electromyography.
