# Supplementary material for: STUB1 stabilizes sirtuin 6 via K63-linked ubiquitination to promote NRF2 deacetylation for enhanced ferroptosis resistance after spinal cord injury
Source: Burns Trauma. 2026 Jul 14;14:tkag047. doi: 10.1093/burnst/tkag047 (PMC13580083; doi:10.1093/burnst/tkag047)
Supplement: Supplementary file 1 [file supplementary_material_tkag047.zip › Figure S4.docx]

**Figure S4**

 **Figure S4: SIRT6 mediates anti-ferroptosis effects by activating NRF2 levels.**

**a** Cell viability in indicated groups analyzed by CCK-8. **b-d** Fe^2+^ concentrations, MDA and GSH in indicated groups were measured (n = 6). *p <.05; **p <.01; ***p <.001. SIRT6:Sirtuin 6;NRF2: Nuclear transcription factor erythroid 2-related factor 2; MDA: Malondialdehyde; GSH: Glutathione.
