# Supplementary material for: STUB1 stabilizes sirtuin 6 via K63-linked ubiquitination to promote NRF2 deacetylation for enhanced ferroptosis resistance after spinal cord injury
Source: Burns Trauma. 2026 Jul 14;14:tkag047. doi: 10.1093/burnst/tkag047 (PMC13580083; doi:10.1093/burnst/tkag047)
Supplement: Supplementary file 1 [file supplementary_material_tkag047.zip › Figure S5.docx]

**Figure S5**

**Figure S5: STUB1 mediates ubiquitination of SIRT6.**

In the presence of MG132, cell lysates were immunoprecipitated with anti-SIRT6 from primary neurons transfected with shNC and shSTUB1, followed by immunoblotting with the indicated antibodies(n=3). SIRT6:Sirtuin 6; STUB1: STIP1 homology and U-box containing protein 1;GAPDH: Glyceraldehyde-3-phosphate Dehydrogenase.
