# Supplementary material for: STUB1 stabilizes sirtuin 6 via K63-linked ubiquitination to promote NRF2 deacetylation for enhanced ferroptosis resistance after spinal cord injury
Source: Burns Trauma. 2026 Jul 14;14:tkag047. doi: 10.1093/burnst/tkag047 (PMC13580083; doi:10.1093/burnst/tkag047)
Supplement: Supplementary file 1 [file supplementary_material_tkag047.zip › Figure S6.docx]

**Figure S6**

**Figure S6:** **STUB1 inhibits ferroptosis and improves electrophysiological function after SCI.**

**a** Representative western blot showing the expression of 4HNE protein in the injured spinal cord at 7 days postinjury (left). Quantification of relative levels of related protein (right, n = 6).**b** Representative images and quantification of EMG recordings of gastrocnemius muscle in mice at 28 days postinjury (n = 6). *p <.05; **p <.01; ***p <.001. 4-HNE:4-hydroxynonenal; GAPDH: Glyceraldehyde-3-phosphate Dehydrogenase; EMG: Electromyography.
