# Supplementary material for: STUB1 stabilizes sirtuin 6 via K63-linked ubiquitination to promote NRF2 deacetylation for enhanced ferroptosis resistance after spinal cord injury
Source: Burns Trauma. 2026 Jul 14;14:tkag047. doi: 10.1093/burnst/tkag047 (PMC13580083; doi:10.1093/burnst/tkag047)
Supplement: Supplementary file 1 [file supplementary_material_tkag047.zip › Figure S7.docx]

**Figure S7**

**Figure S7: STUB1-stabilized SIRT6 results in increased NRF2 transcriptional activity.**

Effects of STUB1 overexpression or SIRT6 knockdown on the mRNA expression levels of NRF2 target genes in neuronal cells (n=3). *p <.05; **p <.01; ***p <.001. STUB1: STIP1 homology and U-box containing protein 1; SIRT6:Sirtuin 6; NRF2: Nuclear transcription factor erythroid 2-related factor 2; HO-1: Heme oxygenase-1; GCLC: Glutamate-Cysteine ligase catalytic subunit; GCLM: Glutamate-Cysteine ligase modifier subunit;NQO1: NADPH quinone dehydrogenase 1.
